# Targeting mitochondrial respiration and the BCL2 family in high‐grade MYC‐associated B‐cell lymphoma

**DOI:** 10.1002/1878-0261.13115

**Published:** 2021-11-11

**Authors:** Giulio Donati, Micol Ravà, Marco Filipuzzi, Paola Nicoli, Laura Cassina, Alessandro Verrecchia, Mirko Doni, Simona Rodighiero, Federica Parodi, Alessandra Boletta, Christopher P. Vellano, Joseph R. Marszalek, Giulio F. Draetta, Bruno Amati

**Affiliations:** ^1^ European Institute of Oncology (IEO)—IRCCS Milan Italy; ^2^ IRCCS San Raffaele Scientific Institute Milan Italy; ^3^ Translational Research to Advance Therapeutics and Innovation in Oncology (TRACTION) Houston TX USA; ^4^ Department of Genomic Medicine University of Texas MD Anderson Cancer Center Houston TX USA; ^5^ Present address: IRCCS San Raffaele Scientific Institute Milan Italy

**Keywords:** BCL2, chemotherapy, DLBCL, Integrated Stress Response, MYC, OxPhos

## Abstract

Multiple molecular features, such as activation of specific oncogenes (e.g., *MYC*, *BCL2*) or a variety of gene expression signatures, have been associated with disease course in diffuse large B‐cell lymphoma (DLBCL), although their relationships and implications for targeted therapy remain to be fully unraveled. We report that MYC activity is closely correlated with—and most likely a driver of—gene signatures related to oxidative phosphorylation (OxPhos) in DLBCL, pointing to OxPhos enzymes, in particular mitochondrial electron transport chain (ETC) complexes, as possible therapeutic targets in high‐grade MYC‐associated lymphomas. In our experiments, indeed, MYC sensitized B cells to the ETC complex I inhibitor IACS‐010759. Mechanistically, IACS‐010759 triggered the integrated stress response (ISR) pathway, driven by the transcription factors ATF4 and CHOP, which engaged the intrinsic apoptosis pathway and lowered the apoptotic threshold in MYC‐overexpressing cells. In line with these findings, the BCL2‐inhibitory compound venetoclax synergized with IACS‐010759 against double‐hit lymphoma (DHL), a high‐grade malignancy with concurrent activation of *MYC* and *BCL2*. In *BCL2*‐negative lymphoma cells, instead, killing by IACS‐010759 was potentiated by the Mcl‐1 inhibitor S63845. Thus, combining an OxPhos inhibitor with select BH3‐mimetic drugs provides a novel therapeutic principle against aggressive, MYC‐associated DLBCL variants.

## Graphical abstract

We show that MYC activity is closely correlated with OxPhos‐associated gene signatures in human B‐cell lymphomas and sensitizes B cells to the mitochondrial complex I inhibitor IACS‐010759. This molecule shows synergy with select BH3‐mimetic drugs in killing cancer cells, and in particular with the BCL2 inhibitor venetoclax against the aggressive double‐hit lymphoma subtype, with promising antitumoral effects in preclinical models.

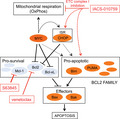

**Abbreviations**
AML: Acute Myeloid Leukemia
ATF4: cyclic AMP‐dependent Transcription Factor‐4
BCL2: B‐Cell Lymphoma 2
BH3: BCL2 homology 3
CCC: *comprehensive consensus clustering*
CHOP: DNA damage‐inducible transcript 3 protein (Ddit3)
COO: *cell‐of‐origin*
DEG: differentially expressed gene
DHL: double‐hit lymphoma
DLBCL: diffuse large B‐cell lymphoma
ETC: electron transport chain
ISR: integrated stress response
Mcl‐1: Myeloid Cell Leukemia‐1
OHT: 4‐hydroxytamoxifen
OxPhos: oxidative phosphorylation
PDX: patient‐derived xenograft
R‐CHOP: rituximab, cyclophosphamide, doxorubicin, vincristine and prednisone

## 1 Introduction

Diffuse large B‐cell lymphoma (DLBCL) is a heterogeneous disease with variable clinical course. While 50–60% of all patients achieve cure with standard R‐CHOP immune‐chemotherapy, others succumb despite aggressive salvage regimens, including high‐dose chemotherapy with autologous stem cell support, allogeneic transplantation, or CAR T‐cell therapy [1]. Multiple genetic and molecular features have been associated with disease course in DLBCL, including translocation and/or overexpression of specific oncogenes (e.g., *MYC*, *BCL2*), mutational profiles, and transcriptome‐based classifiers such as the so‐called *cell‐of‐origin* (COO) and *comprehensive consensus clustering* (CCC) [1, 2, 3, 4, 5]. While *MYC*, *BCL2,* and COO‐associated gene expression signatures are now commonly monitored in the clinic [1], other features have yet to make their way into routine practice, and full elucidation of their clinical relevance is still lacking. Moreover, complex combinatorial arrangements of the above features have far‐reaching implications in disease classification and prognosis, some of which are just beginning to emerge [4, 5, 6].

A series of coincidental observations pointed to a hypothetical relationship between *MYC* and one of the signatures defined in the CCC model, termed ‘OxPhos’ (henceforth CCC‐OxPhos) based on its enrichment for genes involved in Oxidative Phosphorylation [2]. First, *MYC* activation (whether assessed by gene rearrangement, protein expression, or associated gene signatures) and CCC‐OxPhos have both been linked to poor prognosis in DLBCL patients treated with R‐CHOP [2, 3, 7, 8, 9], both behaving independently from the COO classification system [2, 3, 4]. Second, MYC up‐regulates multiple genes involved in mitochondrial transcription and translation [10, 11, 12] and increases the cells’ dependency upon those processes [11, 12], both of which are required for assembly of the electron transport chain (ETC) and oxidative phosphorylation. In line with this principle, tigecycline—an antibiotic that inhibits the mitochondrial ribosome and impairs OxPhos activity [13, 14]—showed increased toxicity toward either MYC‐overexpressing cells [11, 12] or DLBCL cell lines of the CCC‐OxPhos subtype [15]. Yet, whether MYC‐ and OxPhos‐associated gene expression signatures might be related in DLBCL remains to be addressed: we show here that this is indeed the case, with high levels of correlation across multiple patient cohorts.

Altogether, the above observations led us to assess the potential of ETC inhibitors as therapeutic agents against MYC‐associated high‐grade DLBCL. We focused on IACS‐010759 [16], a small‐molecule inhibitor of ETC complex I that showed antitumoral activity in preclinical models of acute myeloid leukemia (AML), chronic lymphocytic leukemia, mantle cell lymphoma and lung cancer [16, 17, 18, 19]. Our data show that IACS‐010759 preferentially induces apoptosis in MYC‐overexpressing cells and cooperates with the BCL2 inhibitor venetoclax in killing *MYC/BCL2* DHL cells, as previously reported for Tigecycline [20]. Furthermore, in BCL2‐negative, *MYC*‐translocated lymphoma cell lines, the cytotoxic activity of IACS was potentiated by the Mcl‐1 inhibitor S63845. Hence, our data point to the possible use of ETC inhibitors in combination with distinct BH3‐mimetic compounds [21] for the treatment of high‐grade DLBCL, in a subtype‐ and patient‐specific manner.

## 2 Materials and methods

### 2.1 Analysis of DLBCL cohorts

For survival and correlation analyses in R‐CHOP‐treated DLBCL patients’ cohorts, we used gene expression data from six publicly available datasets [3, 4, 5, 22, 23, 24]: Data collection methodologies and patient written consents were as provided in the original studies. For RNA‐seq data [3, 5, 24], RNA FastQ files were processed with the same pipeline used for our RNA‐seq data, except for being aligned to hg19; for microarray data [4, 22, 23], probes were matched to the corresponding gene and, for those genes with more than 1 probe set, the mean expression was calculated. In all cases, data were first normalized to Transcripts Per kilobase Million (TPM); then, for each gene, the *z*‐score across all samples was calculated. The Log‐rank test was applied (survminer r package, https://rpkgs.datanovia.com/survminer/index.html, https://cran.r‐project.org/web/packages/survival/index.html) for comparing survival curves of R‐CHOP‐treated patients stratified according to the mean *z*‐score of the expression of the genes belonging to the Hallmark‐MYC‐V1 or OxPhos signatures. For the purpose of computing the linear correlation between signatures within a dataset, outliers were excluded from the calculation. Outliers were identified with the interquartile range (IQR) method as cases were the expression of the signature was not comprised within the following lower and upper boundaries: Lower boundary, 25th quantile – (IQR * 1.5); Upper boundary, 75th quantile + (IQR * 1.5), where IQR = 75th quantile – 25th quantile. Moreover, in order to perform the analysis under stringent conditions, each pairwise correlation was calculated following exclusion of the genes that were shared between the two gene signatures (Table S2).

### 2.2 Cell lines and Xenograft models

The murine lymphoid precursor cell lines Ba/F3 and FL5.12 (RRID: CVCL_0161 and CVCL_0262) [25, 26] and their derivatives were grown in regular RPMI medium (Euroclone, Pero, Italy), which includes 2 mm glutamine and 11 mm glucose, supplemented with 10% fetal bovine serum (FBS) and murine interleukin 3 (PeproTech, Rocky Hill, NJ, USA) at a final concentration of 1 and 2 ng·mL^−1^, respectively. The human lymphoma cell lines DOHH‐2, SU‐DHL‐6, SU‐DHL‐4, Karpas 422, OCI‐LY7, and Ramos (RRID: CVCL_1179, CVCL_2206, CVCL_0539, CVCL_1325, CVCL_1881, and CVCL_0597) were maintained in RPMI medium (including 11 mm glucose) supplemented with 10% FBS. Prior to experiments involving IACS‐010759, all cells were passaged in glucose‐free RPMI‐1640 medium (Thermo Fisher Scientific, Waltham, MA, USA), which includes 2 mm glutamine, supplemented with 10% FBS and 2.75 mm glucose. All cells were incubated at 37 °C in a humidified air atmosphere supplemented with 5% CO2. OCI‐LY7 and Ramos were selected to address differential sensitivity to the Mcl‐1 inhibitor S63845 since they express Mcl‐1 but not BCL2 and are thus resistant to venetoclax [20, 27, 28, 29]. Ba/F3, SU‐DHL‐6, SU‐DHL‐4, and Ramos cell lines were imported from the ATCC repository (https://www.lgcstandards‐atcc.org); DOHH‐2, Karpas 422, and OCI‐LY7 cell lines were imported from the DSMZ repository (https://www.dsmz.de); FL5.12 cells were a gift from Pier Giuseppe Pelicci. All lines were stocked and made available by IEO’s core Tissue Culture facility, where they were also tested for mycoplasma infection.

For the analysis of drug responses *in vivo*, 5 × 10^6^ SU‐DHL‐6 or DOHH‐2 cells were xenografted subcutaneously in irradiated (3 Gray), 8‐week‐old, female CD1‐nude nu/nu mice (Envigo, Indianapolis, IN, USA), and expanded by serial subcutaneous transplantation of tumor fragments. Tumors were allowed to grow for about 2 weeks, followed by exclusion of outliers, randomization of the experimental groups and start of treatments (days 0–11). Tumor volumes were assessed from the start of the treatment every 2 days with a digital caliper and calculated as 1/2 length × width^2^ (mm^3^). The following treatment schemes were used: daily oral gavage with venetoclax and IACS‐010759 for 5 days, followed by two days off and a repeat of the same scheme, for a total of 12 days. Venetoclax was dissolved in 60% Phosal 50 PG (Lipoid, Steinhausen, Switzerland), 30% Polyethylene glycol (Merck, Darmstadt, Germany), 10% Ethanol; IACS‐010759 was suspended in 0.5% methylcellulose (Merck). During the experiment, two animals treated with the highest daily dose of IACS‐010759 (15 mg·kg^−1^), given alone and in combination venetoclax, respectively, showed signs of toxicity, and thus, the treatment was discontinued and the animals were pulled out from the respective group.

The DHL patient‐derived xenograft (PDX) model DFBL‐20954‐V3‐mCLP [30] was obtained from the Dana Farber Cancer Institute Center for Patient Derived Models (CPDM). 1 × 10^6^ cells were xenografted via tail vein injection into 8‐week‐old, male NSG mice (Charles River, Calco, Italy). Tumor engraftment was confirmed 7 days after transplant by whole‐body imaging on an IVIS Lumina III platform following intraperitoneal injection of 150 mg·kg^−1^ XenoLight D‐Luciferin (PerkinElmer, Waltham, MA, USA) and anesthesia with isoflurane. The animals were subsequently randomized in the different experimental groups to start the treatment (day 0–11); the response to treatment was assessed by whole‐body imaging at day 4, day 11, and day 14. The data were analyzed with the Living Image Software, version 4.2 (Caliper Life Sciences, Hopkinton, MA, USA). Radiant efficiency was quantified bilaterally on femurs based on the epifluorescence signal as indicated in the user manual.

Experiments involving animals were done in accordance with the Italian Laws (D.lgs. 26/2014), which enforces Dir. 2010/63/EU (Directive 2010/63/EU of the European Parliament and of the Council of September 22, 2010, on the protection of animals used for scientific purposes), and authorized by the Italian Health Ministry with project nr. 70/2019‐PR. Mice were housed in individually ventilated caging (IVC) systems (Sealsafe Plus, Tecniplast, Buguggiate, Italy), on autoclaved sawdust bedding (Lignocel^®^ 3⁄4; Rettenmaier & Sohne, Ellwangen‐Holzmühle, Germany), provided with autoclaved diet (VRF1 (P), SDS, Witham, UK), and autoclaved water *ad libitum*. Animals were handled and treated in laminar flow hoods (CS5 Evo and BS48, Tecniplast).

### 2.3 Retroviral vectors

Stable expression of exogenous proteins in Ba/F3 and FL5.12 cells was achieved by infection with retroviral vectors. In particular, MycER^T2^ (here MycER) [31] was expressed with the pBabe‐bleo vector [32], human BCL2 with a pMSCV Puromycin vector [33] (a gift from Joseph Opferman), and Ndi1 with PMXS‐Blasticidin (Addgene, Watertwon, CA, USA; plasmid # 72876, a gift from David Sabatini). Transduced cells were selected for one week with the appropriate antibiotic at the following concentrations: zeocin 2 µg·mL^−1^, puromycin 1.5 µg·mL^−1^, blasticidin 5 µg·mL^−1^.

### 2.4 Reagents and kits

L‐aspartic acid (Merck) was added to the growth medium and the pH adjusted to 7.5 with 1 m NaOH before FBS supplementation; hypoxanthine, adenine, and uridine (all from Merck Life Science) were solubilized and added directly to the growth medium. IACS‐010759 [16], venetoclax/ABT‐199 (Carbosynth, Newbury, UK) [34], S63845 (Medchemexpress LCC, Monmouth Junction, NJ, USA) [35], Z‐VAD‐FMK (Santa Cruz Biotechnologies, Santa Cruz, CA, USA) were dissolved in DMSO and added directly to the culture medium; 4‐hydroxytamoxifen (OHT; Merck) was dissolved in ethanol and added directly to the culture medium.

An ADP/ATP Ratio Assay kit (Merck) was used to quantify cellular ADP and ATP, while Caspase‐Glo 3/7 Assay (Promega, Madison, WI, USA) was used to evaluate caspase activity, both according to the manufacturer’s instructions.

### 2.5 Immunoblot analysis

After collection, cells were divided in two aliquots: One was extracted as previously described [36] to be used for quantification by Bradford protein assay (Bio‐Rad Laboratories, Hercules, CA, USA), while the other was directly lysed in Laemmli buffer and denatured at 95 °C for 5 min for subsequent SDS/PAGE. After clearance by centrifugation, 10 µg of proteins from each sample was loaded on an acrylamide gel for SDS/PAGE, followed by transfer on a nitrocellulose membrane and immunoblot with the antibodies listed in Table S3. All data shown are representative of at least *n* = 2 experiments.

### 2.6 Fluorescence microscopy

Nonadherent cells were subjected to cytospin, fixed in 4% paraformaldehyde (PFA), and permeabilized with 0.1% Triton X‐100 for subsequent staining as described previously [37]. Single optical sections were acquired with an SP8 confocal microscope (Leica Microsystems, Wetzlar, Germany) equipped with a 63×/1.4 oil immersion objective lens. For the analysis of apoptotic Figures, cells were stained with 4′,6‐diamidino‐2‐phenylindole (DAPI) and tetramethylrhodamine (TRITC) ‐conjugated Agglutinin (both from Thermo Fisher Scientific). For cytochrome *c* localization, cells were stained overnight at 4 °C with an antibody against cytochrome *c* (clone 7H8.2C12, Thermo Fisher Scientific) and counter‐stained with DAPI. Secondary staining was performed with an Alexa Fluor 488‐conjugated anti‐mouse antibody (Jackson ImmunoResearch, West Grove, PA, USA). The total and cytochrome *c*‐positive cellular areas were calculated from > 20 fields for each sample with an in‐house developed macro for imagej (RRID: SCR_003070). Briefly, the cytochrome *c* signal from each cell in the field of view was segmented using an automatic threshold (Otsu algorithm) and the area of the signal calculated on the binary images. The cytochrome *c* area was then normalized by the total cell area, which was identified on the bright‐field transmission image.

### 2.7 Flow cytometry

Flow cytometry was conducted on a MACSQuant Analyzer (Miltenyi Biotec, Bergisch Gladbach, Germany) and data analyzed with flowjo software (version 10.6.1; BD Biosciences, Franklin Lakes, NK, USA; RRID: SCR_008520). For cell number and viability counts, cells were resuspended in ice‐cold PBS in the presence of 1 µg·mL^−1^ Propidium Iodide (P.I.). Cell viability is given as percentage of live cells on total cells counted, while the proliferation index is defined as the ratio of viable cells in the sample to viable cells in the corresponding IACS‐010759‐untreated control.

For evaluation of apoptotic cell death, cells were stained with APC‐conjugated Annexin V (Thermo Fisher Scientific) and P.I. as previously described [11]. The cytoplasmic release of cytochrome *c* was detected as described [38] with few modifications: Briefly, cells were permeabilized with 0.002% digitonin for 20” under vortexing before fixation in 4% PFA and permeabilization with 0.1% Triton X‐100, for subsequent immunostaining with anti‐cytochrome *c* (clone 6H2.B4, BD Biosciences) overnight at 4 °C. Secondary staining was performed with an Alexa Fluor 647‐conjugated anti‐mouse antibody (Jackson ImmunoResearch).

### 2.8 Cell cycle kinetics

For the analysis of cell cycle progression, cells were incubated for 20 min in the presence of 33 µm bromodeoxyuridine (BrdU, Merck), washed, resuspended, and incubated in fresh medium: at the indicated time‐points (Fig. S2A), aliquots were collected for ethanol‐fixation followed by FITC‐conjugated anti‐BrdU (BD Biosciences) and P.I. staining. Stained samples were analyzed by flow cytometry on a MACSQuant Analyzer.

### 2.9 Oxygen consumption rate (OCR) and respiratory parameters

Oxygen consumption rate was measures on the Seahorse XFe96 Analyzer (Agilent Technologies, Santa Clara, CA, USA) using SeaHorse XF Cell Mito Stress Test, following the manufacturer’s instructions. Briefly, on the day of the assay, cells were counted and attached to 96‐well Seahorse cell culture microplates, precoated with Corning™ Cell‐Tak (Sacco, Cadorago, Italy) according to the manufacturer’s instructions, at a density of 80 000 cells per well. Cells were seeded in at least eight wells per experimental condition, in XF RPMI Medium pH 7.4 with 1 mm HEPES (Agilent), supplemented with 2.75 mm glucose, 1 mm sodium pyruvate, 2 mm L‐glutamine and, where specified, 100 nm OHT, 135 nm IACS‐010759, 100 nm venetoclax. The plates were incubated at 37 °C for 1 h in a non‐CO_2_ incubator. After OCR baseline measurements, oligomycin A, Carbonyl cyanide 4‐(trifluoromethoxy)phenylhydrazone (FCCP), and antimycin A/rotenone were added sequentially to each well, to final concentrations of 1, 1.5, and 0.5 μm, respectively. Results were normalized by cell number, measured at the end of the experiment using the CyQUANT Cell Proliferation Assays (Thermo Fisher Scientific). Data are expressed as pmol of oxygen per minute per arbitrary units (pmol·min^−1^·a.u.^−1^). Respiratory parameters were calculated with the Wave Desktop 2.6 software and shown as mean ± SEM of three independent experiments.

### 2.10 RNA extraction and quantitative PCR analysis

Total cellular RNA was extracted using the Quick‐RNA Miniprep kit (Zymo Research, Irvine, CA, USA) and reverse transcribed with the iSCRIPT cDNA Synthesis Kit (Bio‐Rad Laboratories). 10 ng of cDNA was used as template in each real‐time quantitative PCR (qPCR), performed with fast SyberGreen Master Mix (Thermo Fisher Scientific) on a CFX96 Touch Real‐Time PCR Detection System (Bio‐Rad Laboratories). Primer sequences are provided in Table S4.

### 2.11 RNA library preparation and Next‐Generation Sequencing

Libraries for RNA‐Seq were prepared for each sample from 0.5 μg of total RNA. rRNA removal, RNA‐Seq library preparation, and subsequent analyses were performed as previously described [39] with three biological replicates for each experimental condition. Differentially expressed genes (DEGs) were identified using the bioconductor deseq2 package (RRID: SCR_015687) [40] as genes whose *q*‐value is lower than 0.05. Gene set enrichment analysis (GSEA) was performed using the desktop tool of the Broad Institute (https://www.gsea‐msigdb.org/gsea/index.jsp; RRID: SCR_003199) [41] by querying DEGs for the enrichment of Hallmark Gene Sets from the Hallmark MSigDB collection [42] and CCC DLBCL signatures [2]. Upstream regulator analysis of DEGs was performed with the ingenuity pathway analysis software package (QIAGEN, Venlo, Netherlands; RRID: SCR_008653).

### 2.12 CRISPR‐Cas9 constructs and knockout generation

Gene knockout was obtained by targeting two sites for each gene, selected using the CRISPR Targets Track function from UCSC Genome Browser (https://genome‐euro.ucsc.edu/; RRID: SCR_005780): One was selected close to, and the other ca. 100 bases downstream of the start codon. The genomic sequences targeted by the corresponding sgRNAs are provided in Table S5. Complementary DNA oligonucleotides encompassing the sequence of each sgRNA were annealed and ligated into the PX458 plasmid (Addgene; plasmid # 48138, a gift from Feng Zhang) digested with BbsI, as described [43]. For each construct, 1 µg of DNA was electroporated in 4 × 10^5^ FL5.12 cells using the Neon Transfection System (Thermo Fisher Scientific). After two days, GFP positive cells were sorted with FACSMelody (BD Biosciences) and single clones isolated by limiting dilution. Following in vitro expansion, each clone was tested for recombination by extracting genomic DNA with the QuickExtract DNA Extraction Solution (Illumina) and performing PCR amplification with GoTaq polymerase (Promega) with specific primers for each genomic locus (Table S6). Ablation of the targeted protein in the selected clones was confirmed by immunoblot analysis.

### 2.13 Quantification and statistical analysis

Drug interaction landscapes and delta scores for synergy were based on the ZIP model in SynergyFinder [44]. All other statistical analyses were performed with PRISM 8 (GraphPad Software Inc, La Jolla, CA, USA; RRID: SCR_002798); in cell culture experiments, multiple comparisons of treated groups with the untreated control group were performed by one‐way ANOVA with Dunnett’s test. The numbers of independent biological replicates are indicated in the corresponding Figures. Multiple comparison between groups from *in vivo* tumor growth experiments were performed by one‐way ANOVA with Tukey’s test.

## 3 Results

### 3.1 MYC‐ and OxPhos‐associated gene expression signatures are correlated in DLBCL

**Fig. 1:**
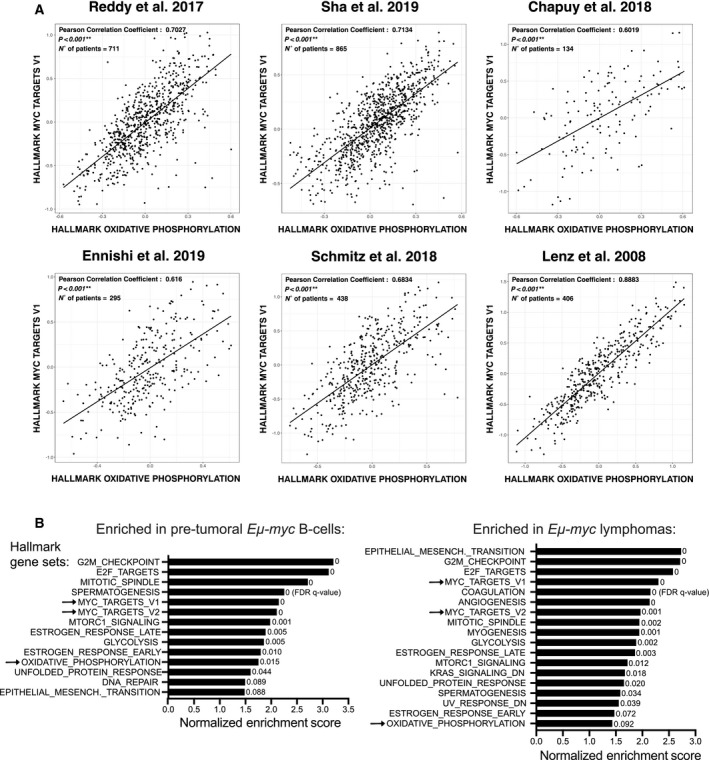
MYC activity positively correlates with the expression of OxPhos‐associated genes. (A) Correlation between the Hallmark‐OxPhos and MYC‐V1 gene sets across gene expression profiles from six independent human DLBCL patient cohorts [3, 4, 5, 22, 23, 24]. The X and Y axes report the mean expression of all genes in the indicated signature, in each patient sample (dots). Black lines represent linear regression fits to the data points. (B) Biological processes enriched during Eµ‐*myc* driven lymphomagenesis. Our previous RNA‐seq data [45] were used to address the enrichment of gene sets from the Hallmark collection and the CCC model [2] in pretumoral Eµ‐*myc* B cells (left) and lymphomas (right), relative to control nontransgenic B cells. The plot shows the enriched genes signatures (FDR *q*‐value < 0.1), ranked according to their normalized enrichment score. Only a subset of the Hallmark‐associated biological pathways, but none of the CCC signatures, reached this threshold. The arrows indicate the MYC target and OxPhos gene signatures.

To address a possible relationship between MYC and OxPhos in DLBCL, we determined the pairwise correlations between four reference signatures, comprising either MYC‐regulated genes (Hallmark‐MYC‐V1 and Hallmark‐MYC‐V2) or genes related to oxidative phosphorylation (Hallmark‐OxPhos and CCC‐OxPhos), in six independent DLBCL datasets [3, 4, 5, 22, 23, 24]. Remarkably, we found a significant positive correlation in all patient cohorts for the MYC‐ and OxPhos‐associated signatures (Table S1), in particular between Hallmark‐MYC‐V1 and Hallmark‐OxPhos (Fig. 1A). Most noteworthy, those two signatures also showed similar relationships with patient survival after R‐CHOP treatment, albeit with inconsistent predictive value among cohorts: significant association with worse outcome in two of the cohorts, loose association in one, and none in three others (Fig. S1A,B). Thus, while variable in terms of their predictive value in R‐CHOP‐treated patients, the MYC‐ and OxPhos‐associated signatures were highly correlated in all DLBCL cohorts.

We then addressed the behavior of the Hallmark and CCC‐derived gene signatures during MYC‐induced lymphomagenesis, based on previous RNA‐seq data in Eµ‐*myc* transgenic mice [45]. Relative to control nontransgenic B cells, either pretumoral Eµ‐*myc* B‐cells or late‐stage lymphomas enriched not only for the MYC signatures, as expected, but also for Hallmark‐OxPhos (albeit with lower significance in the lymphomas, owing most likely to their clonal heterogeneity) [46], while CCC‐OxPhos showed no significant enrichment (Fig. 1B).

Altogether, the above data point to OxPhos as one of the positively regulated gene programs in MYC‐driven lymphomagenesis. We previously reported that the same is true for up‐regulation of the mitochondrial translation machinery [11], which is itself required for OxPhos activity [13, 14, 20]. We thus hypothesized that, like mitochondrial ribosomes [11, 12, 20], OxPhos activity might represent a tractable therapeutic target in MYC‐associated lymphoma.

### 3.2 MYC sensitizes B cells to IACS‐010759‐induced killing through the intrinsic apoptotic pathway

In order to address whether enhanced Myc activity may increase the sensitivity to OxPhos disruption, we transduced two murine B‐cell progenitor lines (FL5.12 and Ba/F3) with retroviral vectors driving constitutive expression of a 4‐hydroxytamoxifen (OHT)‐dependent MycER chimera (hereafter FL^MycER^ and BaF^MycER^). At the phenotypic level, 48 h of OHT treatment had no noticeable impact on either cycle transit times or cell death (Fig. S2A,B), owing most likely to already maximal division rates in the basal state (ca. 12 h) and to the presence of survival factors in the culture medium [47]. Yet, as expected, OHT treatment led to the induction of known MYC‐activated mRNAs, as well as suppression of endogenous *Myc* mRNA and protein (Fig. S2C,D). RNA‐seq profiling in OHT‐treated FL^MycER^ cells revealed that MycER activation caused both up‐ and down‐regulation of discrete sets of differentially expressed genes (DEGs: ca. 1200 each; Fig. S2E), as in other cell types [45]. A search for upstream regulators confirmed that MYC was the main driver of the observed transcriptional changes (Fig. S2F).

**Fig. 2:**
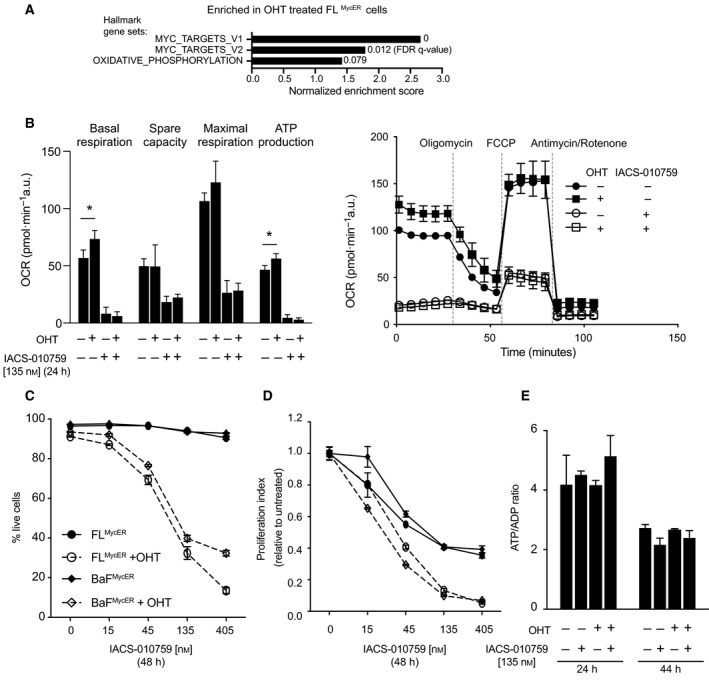
Elevated MYC activity promotes OxPhos and sensitizes B cells to IACS‐010759. (A) Enrichment of MYC target and OxPhos gene sets among upregulated DEGs in FL^MycER^ treated with OHT (100 nm, 72 h). (B‐E) FL^MycER^ of BaF^MycER^ cells were cell pretreated or not with OHT (100 nm, 48 h), followed by IACS‐010759 treatment at the indicated times and concentrations. (B) Left: basal respiration, spare capacity, maximal respiration, and respiration‐coupled ATP production, averaged from three independent mitochondrial stress test profiles in FL^MycER^ cells, treated as indicated (Error bars: SEM; *P < 0.05 from *t*‐test between OHT‐treated cells and their respective controls); a representative profile is shown on the right (Error bars: SD; *n* = 10). (C) Percentage of live cells and (D) Proliferation index in FL^MycER^ and BaF^MycER^ cells. In both panels, OHT‐primed samples treated with ≥ 45 nm IACS‐010759 showed significant differences relative to their controls (*P* < 0.0001); note that in (D) each IACS‐010759‐treated sample was normalized to its untreated control (either with, or without OHT). Error bars: SD (*n* = 3). Cell count and viability were determined by propidium iodide staining. (E) ATP/ADP ratios in FL^MycER^ cells, treated as indicated. Error bars: SD (*n* = 3).

Remarkably, besides the Hallmark‐MYC‐V1 and ‐MYC‐V2 gene sets, MycER‐induced DEGs also enriched for Hallmark‐OxPhos (Fig. 2A). In line with this finding and with the positive effects of MYC on mitochondrial translation [11], OHT treatment induced statistically significant increases in basal respiration and mitochondrial ATP production in FL^MycER^ cells, without significant impact on maximal respiration rate and spare respiratory capacity (Fig. 2B); these effects were specifically due to MycER activation, as they were not observed in parental FL5.12 cells (Fig. S2G). Finally, irrespective of previous OHT treatment, these mitochondrial activities were all suppressed by 24 h of treatment with the ETC complex I inhibitor IACS‐010759 [16] (Fig. 2B).

We thus proceeded to assess the phenotypic consequences of IACS‐010759 treatment in FL^MycER^ and BaF^MycER^ cells. In the absence of OHT, increasing concentrations of IACS‐010759 showed no cytotoxic activity up until 48 h of treatment (Fig. 2C), but a dose‐dependent cytostatic effect (Fig. 2D); instead, pretreatment with OHT markedly sensitized both cell lines to dose‐dependent killing by IACS‐010759. In parental FL5.12 cells, IACS‐010759 was cytostatic regardless of prior OHT treatment (Fig. S3A): hence, sensitization to IACS‐induced cell death was attributable to MycER activation. In order to confirm that the effect of IACS‐010759 was on‐target, we transduced FL^MycER^ cells with a vector expressing the *S. cerevisiae* Ndi1 protein, a IACS‐010759‐resistant ortholog of mammalian ETC complex I that can restore electron transport in complex I‐deficient cells [16, 48]: indeed, as expected, Ndi1 conferred full resistance to IACS‐010759 (Fig. S3B). Finally, OHT promoted killing by another complex I inhibitor, rotenone (Fig. S3C). Hence, MycER activation sensitized cells to inhibition of ETC complex I.

As seen in other cell types [16, 49], the cytotoxic action of IACS was manifest with reduced amounts of glucose (2.75 mm) but not in standard high‐glucose media (11 mm) (Fig. S3D). On this basis, cell killing by IACS‐010759 was previously ascribed to a drop in cellular energy that could be compensated by increasing glycolytic rates [16, 17, 49]. However, energy depletion was unlikely to be the cause of cell death in our system, as neither OHT nor IACS‐010759 treatment impacted the ATP to ADP ratio before the onset of cell death (Fig. 2E). This conservation of the energy balance following IACS‐010759 treatment might be explained by adaptive cellular responses, such as increased glycolysis or suppression of energy‐consuming processes (e. g. cell growth and proliferation). Mitochondrial respiration also sustains aspartate biosynthesis, which in turn is required for anabolic reactions, including protein and nucleotide biosynthesis [50, 51]. In line with this concept, supplementation with aspartate or nucleotide precursors partially bypassed the anti‐proliferative effects of complex I inhibitors in other cell types [16, 50, 51, 52]. However, the same supplements were insufficient to prevent IACS‐010759‐induced arrest and cell death in FL^MycER^ and BaF^MycER^ cells (Fig. S3E), implying that these effects involve additional complex I‐dependent processes.

**Fig. 3:**
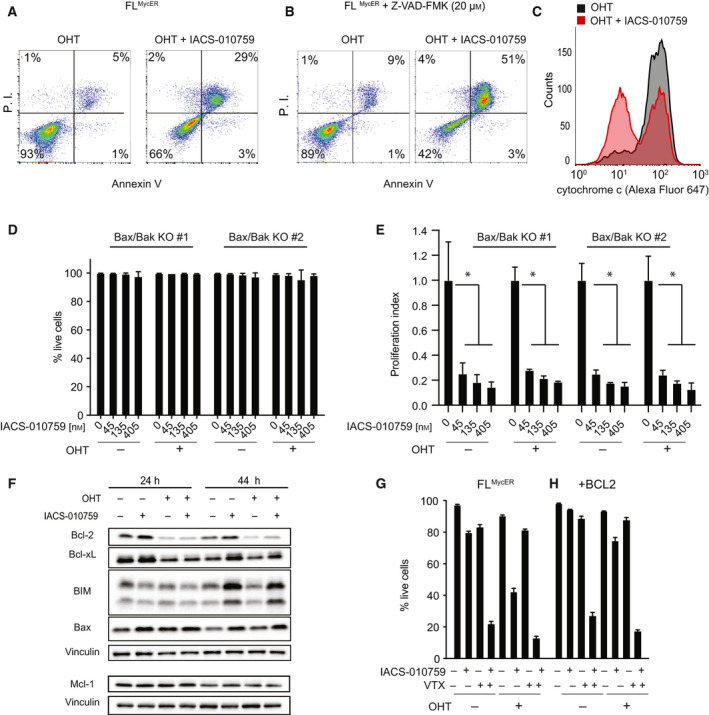
IACS‐010759 activates intrinsic apoptosis in Myc‐overexpressing cells. FL^MycER^ cells were sequentially treated with OHT (100 nm, 48 h), followed by IACS‐010759 (135 nm, unless otherwise indicated; all 48 h). (A) Apoptotic cell death was assayed by FACS analysis of propidium iodide (P.I.) and Annexin V staining (both shown as arbitrary fluorescence units). One representative experiment is shown, from two performed in FL^MycER^ and BaF^MycER^ cells. (B) As in (A) with the addition of 20 µm Z‐VAD‐FMK together with IACS‐010759. (C) Mitochondrial retention of cytochrome *c*, evaluated by anti‐cyt. c staining and FACS analysis of digitonin‐permeabilized FL^MycER^ cells. (D) Percentage of live cells and (E) proliferation index (as defined in Fig. 2) in two independent Bax/Bak double knockout FL^MycER^ clones pretreated or not with OHT, then treated with IACS‐010759 for 48 h. Error bars: SD (*n* = 3). *P < 0.001 vs. IACS‐010759‐untreated control (one‐way ANOVA). (F) Immunoblot analysis of the indicated BCL2‐family members in FL^MycER^ cells pretreated or not with OHT, followed by IACS‐010759 at the indicated times. Vinculin was used as loading control. Note that Mcl‐1 and its own vinculin control (bottom) are from a different experiment, generated during the revision of our manuscript. (G, H) Percentage of live cells in parental FL^MycER^ (G) and FL^MycER^ cells overexpressing BCL2 (H). The cells were pretreated or not with OHT (100 nm, 48 h), then treated for 48 h with IACS‐010759 (135 nm) and/or venetoclax (VTX; 100 nm), as indicated. Error bars: SD (*n* = 3).

Cell death in FL^MycER^ cells treated with OHT and IACS‐010759 presented clear features of apoptosis, such as external membrane exposure of phosphatidylserine (Annexin V staining: Fig. 3A), chromatin condensation and nuclear fragmentation (Fig. S4A), PARP cleavage (Fig. S4B), and caspase activation—the latter suppressed by treatment with the caspase inhibitor Z‐VAD‐FMK (Fig. S4C). Unexpectedly, however, Z‐VAD‐FMK failed to rescue IACS‐010759‐induced killing of OHT‐primed cells (Fig. 3B), implying that caspase activity in not an absolute requirement for apoptosis in this setting, as also observed in other contexts [53, 54].

The apoptotic response can be activated by either the extrinsic or the intrinsic pathway, the latter mediated by permeabilization of the mitochondrial outer membrane, determined by the equilibrium between pro‐ and anti‐apoptotic members of the BCL2 protein family [55, 56]. In particular, the effectors Bax and Bak must disengage from anti‐apoptotic BCL2‐family proteins to form oligomeric pores, through which cytochrome *c* and other proteins are released from the mitochondrial intermembrane space into the cytosol [57]. Indeed, we detected cytochrome *c* release from the mitochondria of OHT‐primed FL^MycER^ cells undergoing IACS‐010759‐induced cell death (Fig. 3C, Fig. S4D). In order to further address the role of intrinsic apoptosis in our model, we derived Bak/Bak‐null FL^MycER^ cell clones through CRISPR‐Cas9 engineering: Remarkably, these knockout cells acquired full resistance to IACS‐010759‐induced cell death (Fig. 3D), while retaining the cytostatic response (Fig. 3E). Hence, IACS‐010759‐mediated cytotoxicity was mediated by the intrinsic apoptotic pathway.

### 3.3 BCL2 acts as a suppressor of IACS‐010759‐induced cell death

Myc is known to perturb the balance between pro‐ and anti‐apoptotic BCL2‐family members by promoting transcription of Bim [58, 59] and Bax [60], while repressing the expression of BCL2 and Bcl‐xL [61, 62], altogether favoring cytochrome *c* release [63]. In order to address whether the increased sensitivity to IACS‐010759 following MycER activation could be ascribed to alterations in balance within the BCL2 family, we monitored protein levels in FL^MycER^ cells following OHT priming and subsequent treatment with IACS‐010759 for 24 and 44 h (both before the actual onset of cell death): Indeed, MycER activation down‐regulated BCL2 and Bcl‐xL, over‐riding a slight increase in these proteins promoted by IACS‐010759 alone (Fig. 3F). In contrast, neither Bim, nor Bax were significantly affected by MycER, while moderately induced by IACS‐010759 (in particular at 44 h: Fig. 3F). Finally, we tested the expression of Mcl‐1, an anti‐apoptotic BCL2‐family protein previously reported to be induced by MYC [64] and suppressed by the ETC complex I inhibitor metformin [65]; within the resolution of these experiments, we did not detect changes in its levels in response to either MycER activation or IACS‐010759 treatment (Fig. 3F bottom).

Based on these results, we hypothesized that reduced expression of BCL2 and Bcl‐xL upon MycER activation may cause the increased sensitivity to IACS‐010759 in FL^MycER^ cells. In support of this concept, the BCL2‐specific inhibitor venetoclax sensitized the cells to IACS‐010759‐induced cell death irrespective of prior MycER activation (Fig. 3G), confirming the importance of endogenous BCL2 in conferring resistance to IACS‐010759. Reciprocally, ectopic expression of BCL2 prevented IACS‐010759‐induced cell death in OHT‐primed cells, a protective effect predictably overridden by venetoclax (Fig. 3H). As reported above for the combination of IACS‐010759 and OHT (Fig. 3B), cell killing by IACS‐010759 and venetoclax was not rescued by Z‐VAD‐FMK (Fig. S4E), further indicating that cell death induced by the intrinsic apoptotic pathway is not dependent on caspase activity in our model.

Previous data indicated that BCL2 can sustain OxPhos activity in leukemic stem cells [66, 67]. Hence, in the above experiments, BCL2 might have acted by boosting mitochondrial respiration and/or protecting it from IACS‐010759‐mediated inhibition. In contrast with this scenario, however, neither overexpression, nor inhibition of BCL2 significantly impacted respiratory activity or its suppression by IACS‐010759 in OHT‐primed FL^MycER^ cells (Fig. S4F,G). Finally, Bax/Bak‐null FL^MycER^ cells remained resistant to IACS‐010759‐induced cytotoxicity even in the presence of venetoclax (Fig. S4H), further emphasizing that BCL2 blocks IACS‐010759‐induced cell death by preventing activation of the intrinsic pathway.

### 3.4 IACS‐010759 treatment activates the Integrated Stress Response

**Fig. 4:**
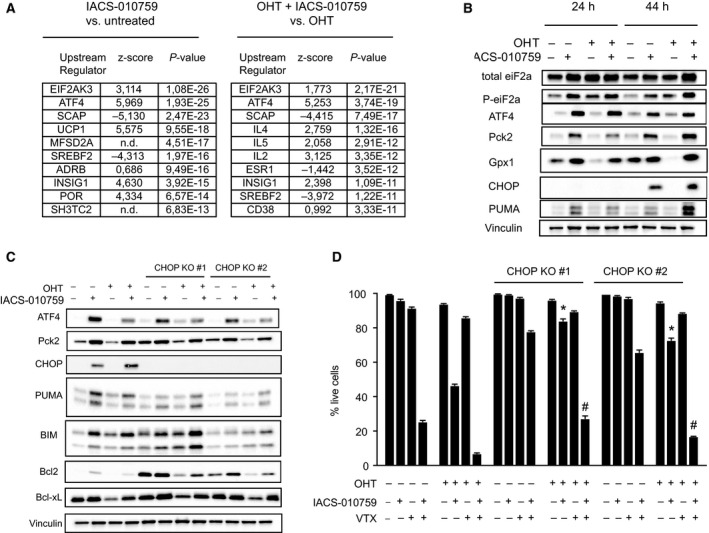
The Integrated Stress Response mediates IACS‐010759‐induced cell death. FL^MycER^ cells were pretreated or not with OHT (100 nm, 48 h), followed by IACS‐010759 (135 nm, 24 h). (A) Gene expression was profiled by RNA‐seq, as defined in the Materials and methods section: the table shows the 10 most significantly enriched Upstream Regulators identified through Ingenuity Pathway Analysis of IACS‐010759‐responsive genes, either with (right) or without OHT (left). n.d.: not determined. *P* values were calculated according to the Fisher’s exact test. (B) Immunoblot analysis of ISR components in FL^MycER^ cells after treatment with IACS‐010759 for 24 or 44 h as indicated. Vinculin was used as loading control. (C) Immunoblot analysis of parental and CHOP‐knockout FL^MycER^ cells after 44 h of IACS‐010759 treatment. (D) Percentage of live cells, confronting parental FL^MycER^ cells with two CHOP‐knockout clones, treated with OHT, followed by IACS‐010759 and/or venetoclax (VTX; 100 nm), as indicated. Error bars: SD (*n* = 3). *P < 0.0001 from *t*‐test between each CHOP KO clone and the parental cells, both treated with OHT and IACS‐101759; ^#^ *P* < 0.0001 from *t*‐test for each CHOP KO clone between cells treated with OHT, IACS‐101759 and VTX, relative to those treated with OHT and IACS‐101759.

In order to characterize possible signals mediating the cytotoxic action of IACS‐010759, we profiled transcriptional changes after 24 h of IACS‐010759 treatment in either OHT‐primed or nonprimed FL^MycER^ cells. Independently from MycER activation, IACS‐010759 elicited extensive transcriptional alterations, with over 1000 DEGs in either OHT‐primed or nonprimed cells and a close correlation between the two responses (Fig. S3E). Upstream regulator analysis identified EIF2AK3 (also known as PERK) and ATF4, two key components of the Integrated Stress Response (ISR) [68], as controllers of the transcriptional response to IACS‐010759 in both OHT‐primed and nonprimed cells (Fig. 4A). Moreover, IACS‐010759 treatment suppressed the expression of genes involved in sterol biosynthesis, which are controlled by the Sterol regulatory element‐binding proteins (SREBPs) and the upstream SCAP‐INSIG1 regulatory circuit [69]. Finally, IACS‐010759 led to the activation of genes associated with the expression of UCP1, which mediates dissipation of the proton gradient across the mitochondrial inner membrane, thus decoupling electron transport from ATP synthesis [70]: this most likely indicates a common cellular response to impaired mitochondrial ATP production, regardless of its cause (i.e., suppression of membrane potential by UCP1, or disruption of the electron transport chain by IACS‐010759; Fig. 2C).

The ISR, the highest ranking IACS‐010759‐regulated program in our analysis (Fig. 4A: EIF2AK3 and ATF4), is an adaptive pathway engaged by diverse stress stimuli that selectively activate one of four different kinases (EIF2AK1‐4), which converge to phosphorylate a specific residue (S51) in the translation factor eIF2a [68]. While repressing general translation, phospho‐eIF2a promotes the translation of a subset of transcripts, including the *ATF4* mRNA. ATF4 and several other ISR‐induced transcriptional regulators, in turn, promote a pro‐survival, stress‐resistance program [68]. Most relevant here, under conditions of severe, unresolved stress, the ISR can also induce pro‐apoptotic factors such as the BH3‐only BCL2‐family proteins Bcl2l11/Bim, Bbc3/PUMA, Hrk, and NOXA [71, 72, 73, 74, 75].

Based on the above, we monitored the status of the ISR pathway in FL^mycER^ cells: Treatment with IACS‐010759, but not OHT, promoted eIF2a phosphorylation and accumulation of ATF4 and several of its target‐gene products, known to be involved in either cell survival (Gpx1, Pck2) or death (Ddit3/CHOP, PUMA, Bim) (Figs 4B and 3F) [68]. Part of these were also significantly upregulated at the mRNA level in our RNA‐seq data (i.e., Pck2 and CHOP), while others were not (Gpx1, PUMA, Bim) (Fig. S3G), possibly reflecting activation of the latter at the translational level, as described in other ISR‐related contexts [76, 77]. Two other known effectors of the ISR, Hrk and NOXA [74], were not expressed in FL^mycER^ cells as judged by our RNA‐seq data, precluding assessment of their possible roles in IACS‐010759‐induced cell death.

In order to assess the contribution of the ISR to the cytotoxic action of IACS‐010759, we used CRISPR‐Cas9 engineering to inactivate the gene encoding CHOP, the main mediator of the pro‐apoptotic branch of the ISR [68]. Previous work suggested that CHOP acts by inducing the BH3‐only proteins PUMA and Bim [71, 72, 73], while suppressing BCL2 expression [78]. However, similar to parental FL^MycER^ cells, CHOP‐null cells showed IACS‐010759‐dependent activation of Bim and PUMA (Fig. 4C). Instead, while still suppressed by MycER activation, BCL2 levels were altered in CHOP knockout cells, with increased amounts in either basal or IACS‐010759‐treated conditions (Fig. 4C). Consistent with the role of BCL2 in IACS‐010759‐induced cell death, CHOP KO cells showed increased resistance to the drug, which was overcome by co‐treatment with venetoclax (Fig. 4D).

### 3.5 IACS‐010759 and venetoclax synergize against *MYC/BCL2* double‐hit lymphoma

**Fig. 5:**
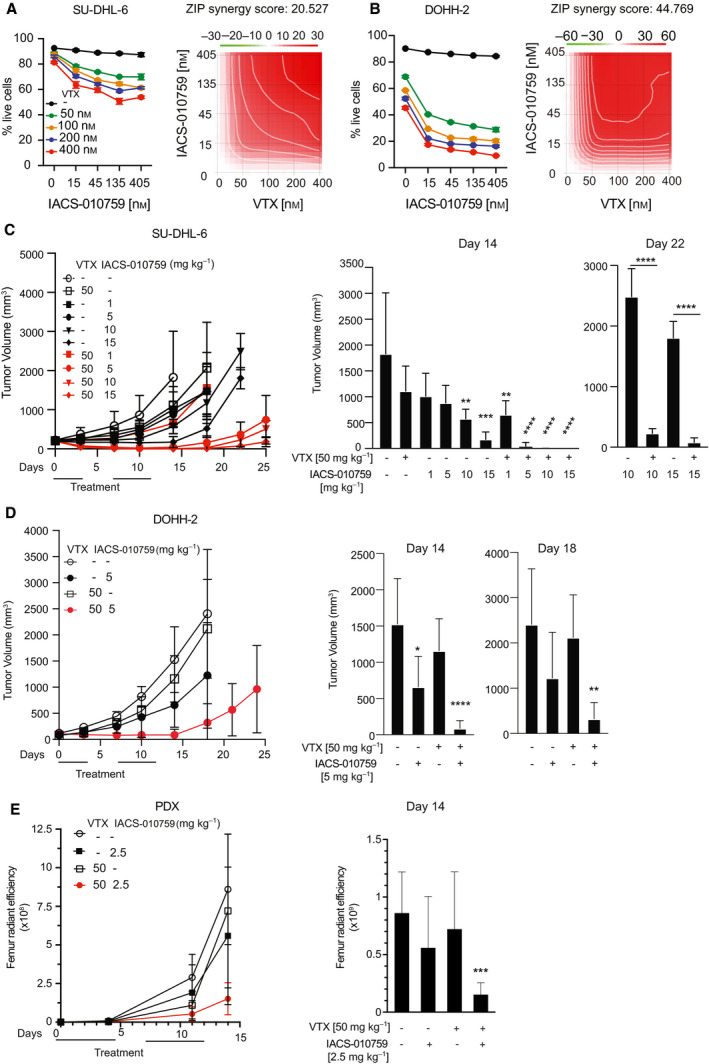
Combinatorial action of IACS‐010759 and BH3‐mimetic compounds against MYC‐associated lymphomas. (A) Left: percentage of live SU‐DHL‐6 cells after 24 h treatment with the indicated concentrations of IACS‐010759 and venetoclax (VTX). Error bars: SD (*n* = 3). Right: drug interaction landscape and synergy score for the two drugs calculated according to the ZIP model. Note that a positive ZIP score (> 10) signifies a synergistic interaction. The landscape identifies the doses at which the drugs either synergize (red) or antagonize each‐other (green)—the latter not observed here. (B) As in (A), for DOHH‐2 cells. (C) Tumor progression in CD1 nude mice bearing subcutaneous SU‐DHL‐6 tumors treated by oral gavage with the indicated daily doses of IACS‐010759 and/or venetoclax. Tumor volumes (mm^3^) were monitored at the indicated time‐points. Error bars: SD; *n* = 5 animals per group. The mean values, standard deviations and statistical significance for individual groups are visualized on the right: *P < 0.05; **P < 0.01; ***P < 0.001; ****P < 0.0001 (one‐way ANOVA relative to the untreated control at day 14, to the VTX‐only group at day 22). (D) As in (C) for subcutaneous DOHH‐2 tumors (all relative to untreated control). (E) Tumor progression in NSG mice injected with the luciferase‐positive PDX line DFBL‐20954‐V3‐mCLP. Tumor development in individual mice was monitored by *in vivo* imaging through quantification of bilateral femur radiant efficiency. After randomization, the animals were subjected to treatment with 2.5 mg·kg^−1^ IACS‐010759 and/or 50 mg·kg^−1^ venetoclax (VTX). Error bars: SD; *n* = 4/5 animals per group. Right: mean values and SD at day 14; ***P < 0.001 (one‐way ANOVA).

The preferential killing of Myc‐overexpressing cells by IACS‐010759 (Fig. 2A) and its suppression by Bcl2 (Fig. 3G,H) were reminiscent of our previous results with Tigecycline [11, 20]. We also reported that Tigecycline and venetoclax cooperated in killing *MYC/BCL2* double‐hit lymphoma cells, and showed synergy against DHL in a preclinical setting [20]. Hence, these observations prompted us to address the potential of combining IACS‐010759 and venetoclax to treat DHL. Indeed, dosing either drug against the other *in vitro* on the human DHL cell lines SU‐DHL‐6 and DOHH‐2 revealed a strong synergy in cell killing (Fig. 5A,B), further substantiated with fixed concentrations of each drug in two other DHL lines (Fig. S5A). It is noteworthy here that as in FL5.12 cells (Fig. 4A,B), IACS‐010759 treatment induced an ISR in DHL cells—albeit to variable levels—as indicated by eIF2a phosphorylation and ATF4 accumulation (Fig. S5B).

In order to address the combinatorial effect of the drugs *in vivo*, CD1 nude mice bearing subcutaneous SU‐DHL‐6 tumors were treated by oral gavage with IACS‐010759 (1–15 mg·kg^−1^) and/or venetoclax (50 mg·kg^−1^), for a total of 10 daily doses over two weeks. While either drug alone or the combination with the lowest dose of IACS‐010759 moderately slowed down tumor growth during the treatment period, combinations with higher doses of IACS‐010759 showed increased efficacy and a marked delay in re‐growth post‐treatment (Fig. 5C), most animals showing either partial or complete tumor regression up to one week after the end of treatment (day 19; Fig. S5C). Similar results were obtained in mice transplanted with DOHH‐2 cells (Fig. 5D). We then monitored the response of the DHL patient‐derived xenograft (PDX) DFBL‐20954‐V3‐mCLP, isolated from a lymphoma refractory to multiple chemotherapy regimens [30]. PDX cells were inoculated by tail vein injection and their growth monitored by whole‐body luminescence in NSG mice: again, IACS‐010759 or venetoclax alone showed little effect, while the combination strongly suppressed tumor growth after the end of the treatment cycles (Fig. 5E, Fig. S6). Altogether, IACS‐010759 and venetoclax showed strong antitumoral activity against DHL in the preclinical setting.

**Fig. 6:**
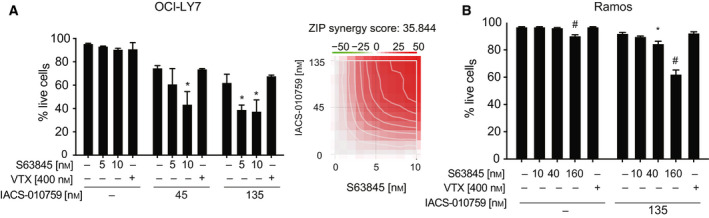
Combinatorial action of IACS‐010759 and the Mcl‐1 inhibitor S63845 in DLBCL and BL cell lines. (A) OCI‐LY7 and (B) Ramos cells were treated for 24 h with IACS‐010759 and either venetoclax or S63845, at the indicated concentrations. The graphs show the percentage of live cells after treatment. Error bars: SD (*n* = 3). *P < 0.01; ^#^ *P* < 0.0001 vs. untreated control (one‐way ANOVA). The heatmap in (A) shows the drug interaction landscape and synergy score for IACS‐010759 and S63845 (as in Fig. 5A).

**Fig. 7:**
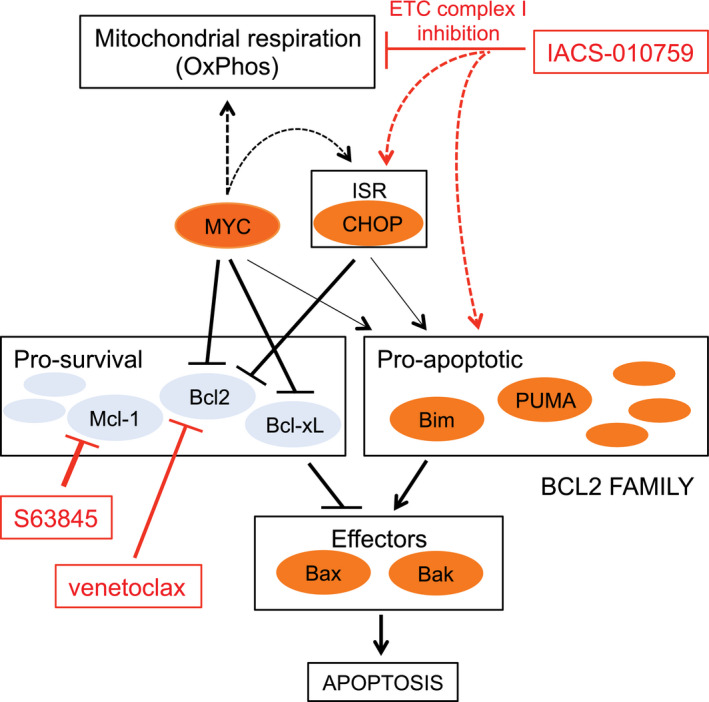
Combinatorial targeting of OxPhos and BCL2‐family proteins in MYC‐associated lymphoma. Schematic summary of the pharmaco‐genetic interactions described in this work. Dashed arrows represent indirect effects; the connections between MYC, the integrated stress response (ISR), and the pro‐apoptotic BCL2 arm (thin arrows) were described in other studies (see text) and may further reinforce the sensitivity of MYC‐overexpressing cells to ETC inhibitors. Among a number of other pathways, MYC supports mitochondrial respiration (Oxphos): inhibition of this process, and in particular of ETC complex I by IACS‐010759 is synthetic‐lethal with MYC, pointing to Oxphos as an important effector in MYC‐induced tumorigenesis. Mechanistically, IACS‐010759 induces apoptosis through activation of the ISR and in particular its pro‐apoptotic effector CHOP and may independently impact BCL2‐family proteins. MYC sensitizes to apoptosis by modulating the expression of BCL2‐family members (and, not shown here, activation of the ARF/p53 pathway). Hence, the ISR and MYC activity converge on the BCL2 family to lower the apoptotic threshold upon IACS‐010759 treatment. This model provides a coherent rationale for the effects reported in this work, including (a) MYC‐induced sensitization of B cells to killing by IACS‐010759 and (b) the cooperative cytotoxic action of IACS‐010759 and BH3‐mimetic compounds, in particular venetoclax in MYC/BCL2 DHL cells, and S63845 in Mcl‐1‐expressing DLBCL and Burkitt’s lymphoma cells.

The above results pointed to a wider therapeutic strategy against other high‐grade lymphomas. In particular, in cases showing translocation of MYC alone, tumor progression and survival may be under the control of distinct anti‐apoptotic BCL2‐family proteins, against which specific pharmacological inhibitors are also available [21]: the latter compounds—rather than venetoclax—may thus show synergy with IACS‐010759 in these tumors. We tested this hypothesis in OCI‐LY7, a MYC‐translocated, BCL2‐negative DLBCL cell line that is fully resistant to venetoclax [20], but expresses Mcl‐1 [29]. Indeed, while IACS‐010759 alone showed substantial toxicity in those cells, this was significantly reinforced by co‐treatment with the Mcl‐1‐specific inhibitor S63845, but not with venetoclax (Fig. 6A). The Burkitt’s lymphoma cell line Ramos, which is also characterized by MYC translocation and expression of Mcl‐1 but not BCL2, showed a similar sensitivity profile (Fig. 6B). Thus, depending upon the cellular context, combination of IACS‐010759 with distinct BH3‐mimetic compounds may allow maximal antitumoral activity (Fig. 7).

## 4 Discussion

Our work points to oxidative phosphorylation (OxPhos) as one of the critical MYC‐activated processes in DLBCL, and as a tractable therapeutic target in high‐grade, MYC‐associated forms of the disease. First, MYC‐ and OxPhos‐related gene signatures were highly correlated in six distinct DLBCL patient cohorts, and were enriched in MYC‐overexpressing mouse B‐cells and lymphomas. Most noteworthy here, MYC may drive OxPhos genes not only at the transcriptional, but also at the translational level in B‐cells [79]. Second, ectopic MYC activity sensitized B‐cells to IACS‐010759, an inhibitor of ETC complex I [16]. Third, IACS‐010759 showed synergy with BH3‐mimetic compounds that inhibit either BCL2 or Mcl‐1, allowing context‐dependent killing of lymphoma cell lines (Fig. 7). Finally, IACS‐010759 and the BCL2 inhibitor venetoclax effectively cooperated against *MYC/BCL2* double‐hit lymphoma in xenograft‐based preclinical models.

It has long been known that MYC overexpression sensitizes diverse cell types to apoptosis in response to environmental or cell‐autonomous stress conditions [47, 63, 80], in a manner that can be counteracted by BCL2 [80, 81]. In line with this concept, ectopic activation of a MycER chimera sensitized two mouse B‐cell lines to killing by IACS‐010759, an effect that was blocked by BCL2 and, reciprocally, exacerbated by venetoclax. Yet, unlike reported in AML cells [66, 67], venetoclax did not suppress OxPhos activity in B‐cells, indicating that it did not directly enhance the inhibitory action of IACS‐010759 on ETC complex I. At the mechanistic level, and as described in other contexts [61, 62], MycER activation suppressed expression of BCL2 and Bcl‐xL. Further analysis confirmed that IACS‐010759 induced cell death through the intrinsic apoptotic pathway, as evidenced by the requirement for the effectors Bax and Bak, and cytoplasmic release of cytochrome *c*. Altogether, as depicted in Fig. 7, MYC promoted OxPhos activity and concomitantly lowered the anti‐apoptotic safeguard provided by BCL2/Bcl‐xL in B‐cells [82], thus sensitizing the cells to IACS‐010759. Our findings are also consistent with a recent study in AML, in which venetoclax and IACS‐010759 synergized to induce apoptosis in AML cells [83]. The MYC‐dependent suppression of both BCL2 and Bcl‐xL also suggests that the combination of IACS‐010759 with the BCL2/Bcl‐xL double inhibitor navitoclax might as well constitute an effective chemotherapy regimen in MYC‐overexpressing tumors, if navitoclax‐associated toxicity is properly managed [84, 85].

We and others have shown that the cytotoxic effects of IACS‐010759 *in vitro* are enhanced by reduced glucose in the medium (Fig. S3C) [16, 49]; therefore, reducing glucose supply to the tumor might improve the efficacy of the drug *in vivo*. In line with this concept, intermittent fasting reduced glycemia and improved the anti‐tumoral activity of a different ETC complex I inhibitor, metformin, in a xenograft model of AML [65]. Whether combining mitochondrial inhibitors, such as IACS‐010759 or metformin, with dietary restriction bears clinical potential against cancer remains to be investigated.

To identify possible effectors of IACS‐010759, we profiled gene expression in B‐cells: this singled out the Integrated Stress Response (ISR) as an IACS‐010759‐induced pathway, independently from MycER activation. The ISR is a composite signaling pathway that mediates protective adaptation to multiple stresses, but concurrently promotes cell death when homeostasis cannot be restored [68]: activation of this pathway in response to OxPhos inhibitors was also reported in AML, multiple myeloma and glioblastoma cells, in which it appeared to relay mainly a pro‐death signal [67, 86, 87]. In line with these observations, elimination of CHOP, which controls the pro‐apoptotic branch of the ISR [68], conferred resistance to IACS‐010759 in our experiments, thus pointing to the ISR as a common effector of OxPhos inhibitors in diverse tumor types. Finally, we shall note here that MYC‐induced oncogenic stress can also activate the ISR [88]: while not observed in our experimental setting, this may further contribute to the sensitivity of MYC‐driven tumors to ETC inhibitors (Fig. 7).

## 5 Conclusions

While IACS‐010759 directly inhibits ETC complex I, thus suppressing OxPhos activity [16], tigecycline achieves the same effect indirectly via inhibition of mitochondrial translation [11, 13], providing a common denominator for their pro‐apoptotic effects on MYC‐overexpressing cells, as well as on DHL cells when combined with venetoclax [20]. Thus, targeting OxPhos (whether with IACS‐010759, tigecycline, etc.) along with select BCL2‐family members [21] may be an effective means to achieve synergy against high‐grade B‐cell lymphomas (Fig. 7). In support of this concept, the BCL2 inhibitor venetoclax potentiated the cytotoxic activity of IACS‐010759 in *MYC/BCL2* DHL cells, while the Mcl‐1 inhibitor S63845 did so in MYC‐translocated, BCL2‐negative lymphoma cell lines. Altogether, our work supports a wider therapeutic concept, in which the status of MYC and BCL2‐family members in individual patients may guide the decision to combine OxPhos inhibitors and select BH3‐mimetics against high‐grade DLBCL, and possibly other refractory malignancies.

## Conflict of interest

The authors declare no conflict of interest.

## Author contributions

GD and MR organized and performed most of the experiments, with technical assistance from PN, AV, and MD. MF performed the computational analyses. FP contributed part of the *in vitro* experiments on cellular drug responses. LC and AB provided support and assistance with the SeaHorse experiments, SR with microscopy, and CPV, JRM, and GFD with the provision of IACS‐010759. GD and BA designed and coordinated the project, and wrote the manuscript.

### Peer Review

The peer review history for this article is available at https://publons.com/publon/10.1002/1878‐0261.13115.

## Supporting information


**Fig. S1**. Variable association of the MYC‐V1 and OxPhos gene signatures with patient survival in DLBCL.
**Fig. S2**. MycER expression and activation in the B‐lymphoid cell lines FL5.12 and Ba/F3.
**Fig. S3**. Effects of MycER activation, IACS‐010759 and other pharmacogenetic interactions in B cells.
**Fig. S4**. Effects of MycER activation, IACS‐010759 and other pharmacogenetic interactions in B cells.
**Fig. S5**. Effects of IACS‐010759 and BH3‐mimetics on human lymphoma cells.
**Fig. S6**. Whole body imaging of PDX‐engrafted mice treated with IACS‐010759 and/or venetoclax.
**Table S1**. MYC‐ and OxPhos‐associated gene signatures are correlated in DLBCL.
**Table S2**. Genes shared among the MYC‐ and OxPhos‐related signatures.
**Table S3**. Antibodies used for immunoblot analysis.
**Table S4**. Primers for mRNA quantification by qPCR.
**Table S5**. Genomic targets of the sgRNAs used for CRISPR‐Cas9 gene knockout.
**Table S6**. Primers for PCR analysis of CRISPR‐Cas9 knockout clones.

## Data Availability

The RNA‐Seq data described in this work are accessible through NCBI's Gene Expression Omnibus (GEO; RRID: SCR_005012) with series accession numbers GSE149073 and GSE51011. RNA‐seq samples and patient’s survival data for Reddy *et al*. [3] and Ennishi *et al*. [24] were made available by the authors and are accessible through the European Genome‐phenome Archive (RRID: SCR_004944) at the European Bioinformatics Institute (https://www.ega‐archive.org/; RRID: SCR_004727) with the accession numbers EGAS00001002606 and EGAS00001002657, respectively. Preprocessed data from Schmitz *et al*. [5] were downloaded from the Genomic Data Commons Data Portal (https://portal.gdc.cancer.gov/; RRID: SCR_014514) with project ID: NCICCR‐DLBCL. The microarray data from Lenz *et al*. [22] (GSE10846), Sha *et al*. [23] (GSE117556), and Chapuy *et al*. [4] (GSE98588) were accessed through NCBI's Gene Expression Omnibus. Access to the EGAS00001002606 dataset [3] was provided by Tushar Dave and Anupama Reddy under an agreement with the Duke University (Durham, NC, USA), and access to the EGAS00001002657 dataset [24] by Stacy Hung, Kal Mann, Theolina Dimitrow, and Christian Steidl under an agreement with BC Cancer (Vancouver, BC, Canada); the results and conclusions reported in this work do not necessarily reflect the opinions or views of BC Cancer. The phs001444 dataset [5] was accessed with a Data Use Certification Agreement from the NIH database for Genotypes and Phenotypes (dbGaP). The PDX model used in this work is covered by a material transfer agreement from the Dana Farber Cancer Institute to Istituto Europeo di Oncologia (DFCI agreement no. A09770).

## References

[1] Liu Y & Barta SK (2019) Diffuse large B‐cell lymphoma: 2019 update on diagnosis, risk stratification, and treatment. Am J Hematol 94, 604–616. 30859597 10.1002/ajh.25460

[2] Monti S , Savage KJ , Kutok JL , Feuerhake F , Kurtin P , Mihm M , Wu B , Pasqualucci L , Neuberg D , Aguiar RC *et al*. (2005) Molecular profiling of diffuse large B‐cell lymphoma identifies robust subtypes including one characterized by host inflammatory response. Blood 105, 1851–1861. 15550490 10.1182/blood-2004-07-2947

[3] Reddy A , Zhang J , Davis NS , Moffitt AB , Love CL , Waldrop A , Leppa S , Pasanen A , Meriranta L , Karjalainen‐Lindsberg ML *et al*. (2017) Genetic and functional drivers of diffuse large B cell lymphoma. Cell 171, 481–494 e15. 28985567 10.1016/j.cell.2017.09.027 PMC5659841

[4] Chapuy B , Stewart C , Dunford AJ , Kim J , Kamburov A , Redd RA , Lawrence MS , Roemer MGM , Li AJ , Ziepert M *et al*. (2018) Molecular subtypes of diffuse large B cell lymphoma are associated with distinct pathogenic mechanisms and outcomes. Nat Med 24, 679–690. 29713087 10.1038/s41591-018-0016-8 PMC6613387

[5] Schmitz R , Wright GW , Huang DW , Johnson CA , Phelan JD , Wang JQ , Roulland S , Kasbekar M , Young RM , Shaffer AL *et al*. (2018) Genetics and pathogenesis of diffuse large B‐cell lymphoma. N Engl J Med 378, 1396–1407. 29641966 10.1056/NEJMoa1801445 PMC6010183

[6] Wright GW , Huang DW , Phelan JD , Coulibaly ZA , Roulland S , Young RM , Wang JQ , Schmitz R , Morin RD , Tang J *et al*. (2020) A probabilistic classification tool for genetic subtypes of diffuse large B cell lymphoma with therapeutic implications. Cancer Cell 37, 551–568.e14. 32289277 10.1016/j.ccell.2020.03.015 PMC8459709

[7] Bisso A , Sabò A & Amati B (2019) MYC in Germinal Center‐derived lymphomas: mechanisms and therapeutic opportunities. Immunol Rev 288, 178–197. 30874346 10.1111/imr.12734

[8] Chiche J , Reverso‐Meinietti J , Mouchotte A , Rubio‐Patino C , Mhaidly R , Villa E , Bossowski JP , Proics E , Grima‐Reyes M , Paquet A *et al*. (2019) GAPDH expression predicts the response to R‐CHOP, the tumor metabolic status, and the response of DLBCL patients to metabolic inhibitors. Cell Metab 29, 1243–1257.e10. 30827861 10.1016/j.cmet.2019.02.002

[9] Carey CD , Gusenleitner D , Chapuy B , Kovach AE , Kluk MJ , Sun HH , Crossland RE , Bacon CM , Rand V , Dal Cin P *et al*. (2015) Molecular classification of MYC‐driven B‐cell lymphomas by targeted gene expression profiling of fixed biopsy specimens. J Mol Diagn 17, 19–30. 25468432 10.1016/j.jmoldx.2014.08.006 PMC4279427

[10] Morrish F & Hockenbery D (2014) MYC and mitochondrial biogenesis. Cold Spring Harb Perspect Med 4, a014225. 24789872 10.1101/cshperspect.a014225 PMC3996374

[11] D'Andrea A , Gritti I , Nicoli P , Giorgio M , Doni M , Conti A , Bianchi V , Casoli L , Sabò A , Mironov A *et al*. (2016) The mitochondrial translation machinery as a therapeutic target in Myc‐driven lymphomas. Oncotarget 7, 72415–72430. 27635472 10.18632/oncotarget.11719 PMC5341918

[12] Oran AR , Adams CM , Zhang XY , Gennaro VJ , Pfeiffer HK , Mellert HS , Seidel HE , Mascioli K , Kaplan J , Gaballa MR *et al*. (2016) Multi‐focal control of mitochondrial gene expression by oncogenic MYC provides potential therapeutic targets in cancer. Oncotarget 7, 72395–72414. 27590350 10.18632/oncotarget.11718 PMC5340124

[13] Skrtic M , Sriskanthadevan S , Jhas B , Gebbia M , Wang X , Wang Z , Hurren R , Jitkova Y , Gronda M , Maclean N *et al*. (2011) Inhibition of mitochondrial translation as a therapeutic strategy for human acute myeloid leukemia. Cancer Cell 20, 674–688. 22094260 10.1016/j.ccr.2011.10.015 PMC3221282

[14] Dong Z , Abbas MN , Kausar S , Yang J , Li L , Tan L & Cui H (2019) Biological functions and molecular mechanisms of antibiotic tigecycline in the treatment of cancers. Int J Mol Sci 20, 3577. 10.3390/ijms20143577 PMC6678986 31336613

[15] Norberg E , Lako A , Chen PH , Stanley IA , Zhou F , Ficarro SB , Chapuy B , Chen L , Rodig S , Shin D *et al*. (2017) Differential contribution of the mitochondrial translation pathway to the survival of diffuse large B‐cell lymphoma subsets. Cell Death Differ 24, 251–262. 27768122 10.1038/cdd.2016.116 PMC5299709

[16] Molina JR , Sun YT , Protopopova M , Gera S , Bandi M , Bristow C , McAfoos T , Morlacchi P , Ackroyd J , Agip ANA *et al*. (2018) An inhibitor of oxidative phosphorylation exploits cancer vulnerability. Nat Med 24, 1036–1046. 29892070 10.1038/s41591-018-0052-4

[17] Vangapandu HV , Alston B , Morse J , Ayres ML , Wierda WG , Keating MJ , Marszalek JR & Gandhi V (2018) Biological and metabolic effects of IACS‐010759, an OxPhos inhibitor, on chronic lymphocytic leukemia cells. Oncotarget 9, 24980–24991. 29861847 10.18632/oncotarget.25166 PMC5982765

[18] Zhang L , Yao Y , Zhang S , Liu Y , Guo H , Ahmed M , Bell T , Zhang H , Han G , Lorence E *et al*. (2019) Metabolic reprogramming toward oxidative phosphorylation identifies a therapeutic target for mantle cell lymphoma. Sci Transl Med 11, eaau1167. 31068440 10.1126/scitranslmed.aau1167

[19] Deribe YL , Sun Y , Terranova C , Khan F , Martinez‐Ledesma J , Gay J , Gao G , Mullinax RA , Khor T , Feng N *et al*. (2018) Mutations in the SWI/SNF complex induce a targetable dependence on oxidative phosphorylation in lung cancer. Nat Med 24, 1047–1057. 29892061 10.1038/s41591-018-0019-5 PMC6650267

[20] Ravà M , D'Andrea A , Nicoli P , Gritti I , Donati G , Doni M , Giorgio M , Olivero D & Amati B (2018) Therapeutic synergy between tigecycline and venetoclax in a preclinical model of MYC/BCL2 double‐hit B cell lymphoma. Sci Transl Med 10, eaan8723. 29386360 10.1126/scitranslmed.aan8723

[21] Adams CM , Clark‐Garvey S , Porcu P & Eischen CM (2018) Targeting the Bcl‐2 family in B cell lymphoma. Front Oncol 8, 636. 30671383 10.3389/fonc.2018.00636 PMC6331425

[22] Lenz G , Wright GW , Emre NC , Kohlhammer H , Dave SS , Davis RE , Carty S , Lam LT , Shaffer AL , Xiao W *et al*. (2008) Molecular subtypes of diffuse large B‐cell lymphoma arise by distinct genetic pathways. Proc Natl Acad Sci USA 105, 13520–13525. 18765795 10.1073/pnas.0804295105 PMC2533222

[23] Sha C , Barrans S , Cucco F , Bentley MA , Care MA , Cummin T , Kennedy H , Thompson JS , Uddin R , Worrillow L *et al*. (2019) Molecular high‐grade B‐cell lymphoma: defining a poor‐risk group that requires different approaches to therapy. J Clin Oncol 37, 202–212. 30523719 10.1200/JCO.18.01314 PMC6338391

[24] Ennishi D , Jiang A , Boyle M , Collinge B , Grande BM , Ben‐Neriah S , Rushton C , Tang J , Thomas N , Slack GW *et al*. (2019) Double‐hit gene expression signature defines a distinct subgroup of germinal center B‐cell‐like diffuse large B‐cell lymphoma. J Clin Oncol 37, 190–201. 30523716 10.1200/JCO.18.01583 PMC6804880

[25] Palacios R & Steinmetz M (1985) Il‐3‐dependent mouse clones that express B‐220 surface antigen, contain Ig genes in germ‐line configuration, and generate B lymphocytes in vivo. Cell 41, 727–734. 3924409 10.1016/s0092-8674(85)80053-2

[26] Mckearn JP , Mccubrey J & Fagg B (1985) Enrichment of hematopoietic precursor cells and cloning of multipotential lymphocyte‐B precursors. Proc Natl Acad Sci USA 82, 7414–7418. 3933007 10.1073/pnas.82.21.7414 PMC391355

[27] Johnson‐Farley N , Veliz J , Bhagavathi S & Bertino JR (2015) ABT‐199, a BH3 mimetic that specifically targets Bcl‐2, enhances the antitumor activity of chemotherapy, bortezomib and JQ1 in “double hit” lymphoma cells. Leuk Lymphoma 56, 2146–2152. 25373508 10.3109/10428194.2014.981172

[28] Stolz C , Hess G , Hahnel PS , Grabellus F , Hoffarth S , Schmid KW & Schuler M (2008) Targeting Bcl‐2 family proteins modulates the sensitivity of B‐cell lymphoma to rituximab‐induced apoptosis. Blood 112, 3312–3321. 18689543 10.1182/blood-2007-11-124487

[29] Klanova M , Andera L , Brazina J , Svadlenka J , Benesova S , Soukup J , Prukova D , Vejmelkova D , Jaksa R , Helman K *et al*. (2016) Targeting of BCL2 family proteins with ABT‐199 and homoharringtonine reveals BCL2‐ and MCL1‐dependent subgroups of diffuse large B‐cell lymphoma. Clin Cancer Res 22, 1138–1149. 26467384 10.1158/1078-0432.CCR-15-1191

[30] Townsend EC , Murakami MA , Christodoulou A , Christie AL , Koster J , DeSouza TA , Morgan EA , Kallgren SP , Liu H , Wu SC *et al*. (2016) The public repository of xenografts enables discovery and randomized phase II‐like trials in mice. Cancer Cell 29, 574–586. 27070704 10.1016/j.ccell.2016.03.008 PMC5177991

[31] Littlewood TD , Hancock DC , Danielian PS , Parker MG & Evan GI (1995) A modified oestrogen receptor ligand‐binding domain as an improved switch for the regulation of heterologous proteins. Nucleic Acids Res 23, 1686–1690. 7784172 10.1093/nar/23.10.1686 PMC306922

[32] Morgenstern JP & Land H (1990) Advanced mammalian gene transfer: high titre retroviral vectors with multiple drug selection markers and a complementary helper‐free packaging cell line. Nucleic Acids Res 18, 3587–3596. 2194165 10.1093/nar/18.12.3587 PMC331014

[33] Koss B , Ryan J , Budhraja A , Szarama K , Yang X , Bathina M , Cardone MH , Nikolovska‐Coleska Z , Letai A & Opferman JT (2016) Defining specificity and on‐target activity of BH3‐mimetics using engineered B‐ALL cell lines. Oncotarget 7, 11500–11511. 26862853 10.18632/oncotarget.7204 PMC4905489

[34] Souers AJ , Leverson JD , Boghaert ER , Ackler SL , Catron ND , Chen J , Dayton BD , Ding H , Enschede SH , Fairbrother WJ *et al*. (2013) ABT‐199, a potent and selective BCL‐2 inhibitor, achieves antitumor activity while sparing platelets. Nat Med 19, 202–208. 23291630 10.1038/nm.3048

[35] Kotschy A , Szlavik Z , Murray J , Davidson J , Maragno AL , Le Toumelin‐Braizat G , Chanrion M , Kelly GL , Gong JN , Moujalled DM *et al*. (2016) The MCL1 inhibitor S63845 is tolerable and effective in diverse cancer models. Nature 538, 477–482. 27760111 10.1038/nature19830

[36] Donati G , Peddigari S , Mercer CA & Thomas G (2013) 5S ribosomal RNA is an essential component of a nascent ribosomal precursor complex that regulates the Hdm2‐p53 checkpoint. Cell Rep 4, 87–98. 23831031 10.1016/j.celrep.2013.05.045 PMC3928573

[37] Sanchez‐Arevalo Lobo VJ , Doni M , Verrecchia A , Sanulli S , Faga G , Piontini A , Bianchi M , Conacci‐Sorrell M , Mazzarol G , Peg V *et al*. (2013) Dual regulation of Myc by Abl. Oncogene 32, 5261–5271. 23318434 10.1038/onc.2012.621 PMC3914638

[38] Campos CB , Paim BA , Cosso RG , Castilho RF , Rottenberg H & Vercesi AE (2006) Method for monitoring of mitochondrial cytochrome *c* release during cell death: immunodetection of cytochrome *c* by flow cytometry after selective permeabilization of the plasma membrane. Cytometry A 69, 515–523. 16680678 10.1002/cyto.a.20273

[39] Tesi A , de Pretis S , Furlan M , Filipuzzi M , Morelli MJ , Andronache A , Doni M , Verrecchia A , Pelizzola M , Amati B *et al*. (2019) An early Myc‐dependent transcriptional program orchestrates cell growth during B‐cell activation. EMBO Rep 20, e47987. 31334602 10.15252/embr.201947987 PMC6726900

[40] Love MI , Huber W & Anders S (2014) Moderated estimation of fold change and dispersion for RNA‐seq data with DESeq2. Genome Biol 15, 550. 25516281 10.1186/s13059-014-0550-8 PMC4302049

[41] Subramanian A , Tamayo P , Mootha VK , Mukherjee S , Ebert BL , Gillette MA , Paulovich A , Pomeroy SL , Golub TR , Lander ES *et al*. (2005) Gene set enrichment analysis: a knowledge‐based approach for interpreting genome‐wide expression profiles. Proc Natl Acad Sci USA 102, 15545–15550. 16199517 10.1073/pnas.0506580102 PMC1239896

[42] Liberzon A , Birger C , Thorvaldsdottir H , Ghandi M , Mesirov JP & Tamayo P (2015) The Molecular Signatures Database (MSigDB) hallmark gene set collection. Cell Syst 1, 417–425. 26771021 10.1016/j.cels.2015.12.004 PMC4707969

[43] Ran FA , Hsu PD , Wright J , Agarwala V , Scott DA & Zhang F (2013) Genome engineering using the CRISPR‐Cas9 system. Nat Protoc 8, 2281–2308. 24157548 10.1038/nprot.2013.143 PMC3969860

[44] Ianevski A , He L , Aittokallio T & Tang J (2017) SynergyFinder: a web application for analyzing drug combination dose‐response matrix data. Bioinformatics 33, 2413–2415. 28379339 10.1093/bioinformatics/btx162 PMC5554616

[45] Sabò A , Kress TR , Pelizzola M , de Pretis S , Gorski MM , Tesi A , Morelli MJ , Bora P , Doni M , Verrecchia A *et al*. (2014) Selective transcriptional regulation by Myc in cellular growth control and lymphomagenesis. Nature 511, 488–492. 25043028 10.1038/nature13537 PMC4110711

[46] Schleich K , Kase J , Dorr JR , Trescher S , Bhattacharya A , Yu Y , Wailes EM , Fan DNY , Lohneis P , Milanovic M *et al*. (2020) H3K9me3‐mediated epigenetic regulation of senescence in mice predicts outcome of lymphoma patients. Nat Commun 11, 3651. 32686676 10.1038/s41467-020-17467-z PMC7371731

[47] Evan GI , Wyllie AH , Gilbert CS , Littlewood TD , Land H , Brooks M , Waters CM , Penn LZ & Hancock DC (1992) Induction of apoptosis in fibroblasts by c‐myc protein. Cell 69, 119–128. 1555236 10.1016/0092-8674(92)90123-t

[48] Seo BB , Kitajima‐Ihara T , Chan EKL , Scheffler IE , Matsuno‐Yagi A & Yagi T (1998) Molecular remedy of complex I defects: rotenone‐insensitive internal NADH‐quinone oxidoreductase of *Saccharomyces cerevisiae* mitochondria restores the NADH oxidase activity of complex I‐deficient mammalian cells. Proc Natl Acad Sci USA 95, 9167–9171. 9689052 10.1073/pnas.95.16.9167 PMC21310

[49] Naguib A , Mathew G , Reczek CR , Watrud K , Ambrico A , Herzka T , Salas IC , Lee MF , El‐Amine N , Zheng W *et al*. (2018) Mitochondrial complex I inhibitors expose a vulnerability for selective killing of Pten‐null cells. Cell Rep 23, 58–67. 29617673 10.1016/j.celrep.2018.03.032 PMC6003704

[50] Birsoy K , Wang T , Chen WW , Freinkman E , Abu‐Remaileh M & Sabatini DM (2015) An essential role of the mitochondrial electron transport chain in cell proliferation is to enable aspartate synthesis. Cell 162, 540–551. 26232224 10.1016/j.cell.2015.07.016 PMC4522279

[51] Sullivan LB , Gui DY , Hosios AM , Bush LN , Freinkman E & Vander Heiden MG (2015) Supporting aspartate biosynthesis is an essential function of respiration in proliferating cells. Cell 162, 552–563. 26232225 10.1016/j.cell.2015.07.017 PMC4522278

[52] Sullivan LB , Luengo A , Danai LV , Bush LN , Diehl FF , Hosios AM , Lau AN , Elmiligy S , Malstrom S , Lewis CA *et al*. (2018) Aspartate is an endogenous metabolic limitation for tumour growth. Nat Cell Biol 20, 782–788. 29941931 10.1038/s41556-018-0125-0 PMC6051729

[53] Kroemer G & Martin SJ (2005) Caspase‐independent cell death. Nat Med 11, 725–730. 16015365 10.1038/nm1263

[54] Tait SW & Green DR (2013) Mitochondrial regulation of cell death. Cold Spring Harb Perspect Biol 5, a008706. 24003207 10.1101/cshperspect.a008706 PMC3753705

[55] Czabotar PE , Lessene G , Strasser A & Adams JM (2014) Control of apoptosis by the BCL‐2 protein family: implications for physiology and therapy. Nat Rev Mol Cell Biol 15, 49–63. 24355989 10.1038/nrm3722

[56] Kalkavan H & Green DR (2018) MOMP, cell suicide as a BCL‐2 family business. Cell Death Differ 25, 46–55. 29053143 10.1038/cdd.2017.179 PMC5729535

[57] Luna‐Vargas MPA & Chipuk JE (2016) Physiological and pharmacological control of BAK, BAX, and beyond. Trends Cell Biol 26, 906–917. 27498846 10.1016/j.tcb.2016.07.002 PMC5118054

[58] Campone M , Noel B , Couriaud C , Grau M , Guillemin Y , Gautier F , Gouraud W , Charbonnel C , Campion L , Jezequel P *et al*. (2011) c‐Myc dependent expression of pro‐apoptotic Bim renders HER2‐overexpressing breast cancer cells dependent on anti‐apoptotic Mcl‐1. Mol Cancer 10, 110. 21899728 10.1186/1476-4598-10-110 PMC3175201

[59] Muthalagu N , Junttila MR , Wiese KE , Wolf E , Morton J , Bauer B , Evan GI , Eilers M & Murphy DJ (2014) BIM is the primary mediator of MYC‐induced apoptosis in multiple solid tissues. Cell Rep 8, 1347–1353. 25176652 10.1016/j.celrep.2014.07.057 PMC4231288

[60] Mitchell KO , Ricci MS , Miyashita T , Dicker DT , Jin Z , Reed JC & El‐Deiry WS (2000) Bax is a transcriptional target and mediator of c‐myc‐induced apoptosis. Cancer Res 60, 6318–6325. 11103792

[61] Eischen CM , Woo D , Roussel MF & Cleveland JL (2001) Apoptosis triggered by Myc‐induced suppression of Bcl‐X(L) or Bcl‐2 is bypassed during lymphomagenesis. Mol Cell Biol 21, 5063–5070. 11438662 10.1128/MCB.21.15.5063-5070.2001 PMC87232

[62] Maclean KH , Keller UB , Rodriguez‐Galindo C , Nilsson JA & Cleveland JL (2003) c‐Myc augments gamma irradiation‐induced apoptosis by suppressing Bcl‐XL. Mol Cell Biol 23, 7256–7270. 14517295 10.1128/MCB.23.20.7256-7270.2003 PMC230315

[63] Juin P , Hueber AO , Littlewood T & Evan G (1999) c‐Myc‐induced sensitization to apoptosis is mediated through cytochrome *c* release. Genes Dev 13, 1367–1381. 10364155 10.1101/gad.13.11.1367 PMC316765

[64] Labisso WL , Wirth M , Stojanovic N , Stauber RH , Schnieke A , Schmid RM , Kramer OH , Saur D & Schneider G (2012) MYC directs transcription of MCL1 and eIF4E genes to control sensitivity of gastric cancer cells toward HDAC inhibitors. Cell Cycle 11, 1593–1602. 22456335 10.4161/cc.20008

[65] Elgendy M , Ciro M , Hosseini A , Weiszmann J , Mazzarella L , Ferrari E , Cazzoli R , Curigliano G , DeCensi A , Bonanni B *et al*. (2019) Combination of hypoglycemia and metformin impairs tumor metabolic plasticity and growth by modulating the PP2A‐GSK3beta‐MCL‐1 axis. Cancer Cell 35, 798–815 e5. 31031016 10.1016/j.ccell.2019.03.007

[66] Lagadinou ED , Sach A , Callahan K , Rossi RM , Neering SJ , Minhajuddin M , Ashton JM , Pei S , Grose V , O'Dwyer KM *et al*. (2013) BCL‐2 inhibition targets oxidative phosphorylation and selectively eradicates quiescent human leukemia stem cells. Cell Stem Cell 12, 329–341. 23333149 10.1016/j.stem.2012.12.013 PMC3595363

[67] Sharon D , Cathelin S , Mirali S , Di Trani JM , Yanofsky DJ , Keon KA , Rubinstein JL , Schimmer AD , Ketela T & Chan SM (2019) Inhibition of mitochondrial translation overcomes venetoclax resistance in AML through activation of the integrated stress response. Sci Transl Med 11, eaax2863. 31666400 10.1126/scitranslmed.aax2863

[68] Pakos‐Zebrucka K , Koryga I , Mnich K , Ljujic M , Samali A & Gorman AM (2016) The integrated stress response. EMBO Rep 17, 1374–1395. 27629041 10.15252/embr.201642195 PMC5048378

[69] McPherson R & Gauthier A (2004) Molecular regulation of SREBP function: the Insig‐SCAP connection and isoform‐specific modulation of lipid synthesis. Biochem Cell Biol 82, 201–211. 15052338 10.1139/o03-090

[70] Klaus S , Casteilla L , Bouillaud F & Ricquier D (1991) The uncoupling protein UCP: a membraneous mitochondrial ion carrier exclusively expressed in brown adipose tissue. Int J Biochem 23, 791–801. 1773883 10.1016/0020-711x(91)90062-r

[71] Puthalakath H , O'Reilly LA , Gunn P , Lee L , Kelly PN , Huntington ND , Hughes PD , Michalak EM , McKimm‐Breschkin J , Motoyama N *et al*. (2007) ER stress triggers apoptosis by activating BH3‐only protein Bim. Cell 129, 1337–1349. 17604722 10.1016/j.cell.2007.04.027

[72] Galehdar Z , Swan P , Fuerth B , Callaghan SM , Park DS & Cregan SP (2010) Neuronal apoptosis induced by endoplasmic reticulum stress is regulated by ATF4‐CHOP‐mediated induction of the Bcl‐2 homology 3‐only member PUMA. J Neurosci 30, 16938–16948. 21159964 10.1523/JNEUROSCI.1598-10.2010 PMC6634926

[73] Ghosh AP , Klocke BJ , Ballestas ME & Roth KA (2012) CHOP potentially co‐operates with FOXO3a in neuronal cells to regulate PUMA and BIM expression in response to ER stress. PLoS One 7, e39586. 22761832 10.1371/journal.pone.0039586 PMC3386252

[74] Pike LR , Phadwal K , Simon AK & Harris AL (2012) ATF4 orchestrates a program of BH3‐only protein expression in severe hypoxia. Mol Biol Rep 39, 10811–10822. 23090478 10.1007/s11033-012-1975-3

[75] Wali JA , Rondas D , McKenzie MD , Zhao Y , Elkerbout L , Fynch S , Gurzov EN , Akira S , Mathieu C , Kay TW *et al*. (2014) The proapoptotic BH3‐only proteins Bim and Puma are downstream of endoplasmic reticulum and mitochondrial oxidative stress in pancreatic islets in response to glucotoxicity. Cell Death Dis 5, e1124. 24625983 10.1038/cddis.2014.88 PMC3973197

[76] Albert AE , Adua SJ , Cai WL , Arnal‐Estape A , Cline GW , Liu Z , Zhao M , Cao PD , Mariappan M & Nguyen DX (2019) Adaptive protein translation by the integrated stress response maintains the proliferative and migratory capacity of lung adenocarcinoma cells. Mol Cancer Res 17, 2343–2355. 31551255 10.1158/1541-7786.MCR-19-0245 PMC6938689

[77] Sidrauski C , McGeachy AM , Ingolia NT & Walter P (2015) The small molecule ISRIB reverses the effects of eIF2alpha phosphorylation on translation and stress granule assembly. eLife 4, e05033. 10.7554/eLife.05033 PMC4341466 25719440

[78] McCullough KD , Martindale JL , Klotz LO , Aw TY & Holbrook NJ (2001) Gadd153 sensitizes cells to endoplasmic reticulum stress by down‐regulating Bcl2 and perturbing the cellular redox state. Mol Cell Biol 21, 1249–1259. 11158311 10.1128/MCB.21.4.1249-1259.2001 PMC99578

[79] Singh K , Lin J , Zhong Y , Burcul A , Mohan P , Jiang M , Sun L , Yong‐Gonzalez V , Viale A , Cross JR *et al*. (2019) c‐MYC regulates mRNA translation efficiency and start‐site selection in lymphoma. J Exp Med 216, 1509–1524. 31142587 10.1084/jem.20181726 PMC6605752

[80] Bissonnette RP , Echeverri F , Mahboubi A & Green DR (1992) Apoptotic cell death induced by c‐myc is inhibited by bcl‐2. Nature 359, 552–554. 1406975 10.1038/359552a0

[81] Fanidi A , Harrington EA & Evan GI (1992) Cooperative interaction between c‐myc and bcl‐2 proto‐oncogenes. Nature 359, 554–556. 1406976 10.1038/359554a0

[82] Marsden VS & Strasser A (2003) Control of apoptosis in the immune system: Bcl‐2, BH3‐only proteins and more. Annu Rev Immunol 21, 71–105. 12414721 10.1146/annurev.immunol.21.120601.141029

[83] Liu F , Kalpage HA , Wang D , Edwards H , Huttemann M , Ma J , Su Y , Carter J , Li X , Polin L *et al*. (2020) Cotargeting of mitochondrial complex I and Bcl‐2 shows antileukemic activity against acute myeloid leukemia cells reliant on oxidative phosphorylation. Cancers (Basel) 12, 2400. 10.3390/cancers12092400 PMC7564145 32847115

[84] Roberts AW , Seymour JF , Brown JR , Wierda WG , Kipps TJ , Khaw SL , Carney DA , He SZ , Huang DC , Xiong H *et al*. (2012) Substantial susceptibility of chronic lymphocytic leukemia to BCL2 inhibition: results of a phase I study of navitoclax in patients with relapsed or refractory disease. J Clin Oncol 30, 488–496. 22184378 10.1200/JCO.2011.34.7898 PMC4979082

[85] Wilson WH , O'Connor OA , Czuczman MS , LaCasce AS , Gerecitano JF , Leonard JP , Tulpule A , Dunleavy K , Xiong H , Chiu YL *et al*. (2010) Navitoclax, a targeted high‐affinity inhibitor of BCL‐2, in lymphoid malignancies: a phase 1 dose‐escalation study of safety, pharmacokinetics, pharmacodynamics, and antitumour activity. Lancet Oncol 11, 1149–1159. 21094089 10.1016/S1470-2045(10)70261-8 PMC3025495

[86] Bajpai R , Sharma A , Achreja A , Edgar CL , Wei C , Siddiqa AA , Gupta VA , Matulis SM , McBrayer SK , Mittal A *et al*. (2020) Electron transport chain activity is a predictor and target for venetoclax sensitivity in multiple myeloma. Nat Commun 11, 1228. 32144272 10.1038/s41467-020-15051-z PMC7060223

[87] Shi Y , Lim SK , Liang Q , Iyer SV , Wang HY , Wang Z , Xie X , Sun D , Chen YJ , Tabar V *et al*. (2019) Gboxin is an oxidative phosphorylation inhibitor that targets glioblastoma. Nature 567, 341–346. 30842654 10.1038/s41586-019-0993-x PMC6655586

[88] Tameire F , Verginadis II , Leli NM , Polte C , Conn CS , Ojha R , Salas Salinas C , Chinga F , Monroy AM , Fu W *et al*. (2019) ATF4 couples MYC‐dependent translational activity to bioenergetic demands during tumour progression. Nat Cell Biol 21, 889–899. 31263264 10.1038/s41556-019-0347-9 PMC6608727

