# Supplementary material for: Targeting mitochondrial respiration and the BCL2 family in high‐grade MYC‐associated B‐cell lymphoma
Source: Mol Oncol. 2021 Nov 11;16(5):1132–52. doi: 10.1002/1878-0261.13115 (PMC8895457; doi:10.1002/1878-0261.13115)
Supplement: Supplementary file 1 — Fig. S1. Variable association of the MYC‐V1 and OxPhos gene signatures with patient survival in DLBCL. Fig. S2. MycER expression and activation in the B‐lymphoid cell lines FL5.12 and Ba/F3. Fig. S3. Effects of MycER activation, IACS‐010759 and other pharmacogenetic interactions in B cells. Fig. S4. Effects of MycER activation, IACS‐010759 and other pharmacogenetic interactions in B cells. Fig. S5. Effects of IACS‐010759 and BH3‐mimetics on human lymphoma cells. Fig. S6. Whole body imaging of PDX‐engrafted mice treated with IACS‐010759 and/or venetoclax. Table S1. MYC‐ and OxPhos‐associated gene signatures are correlated in DLBCL. Table S2. Genes shared among the MYC‐ and OxPhos‐related signatures. Table S3. Antibodies used for immunoblot analysis. Table S4. Primers for mRNA quantification by qPCR. Table S5. Genomic targets of the sgRNAs used for CRISPR‐Cas9 gene knockout. Table S6. Primers for PCR analysis of CRISPR‐Cas9 knockout clones. [file MOL2-16-1132-s001.docx]

*Supplementary Materials for:*

Targeting mitochondrial respiration and the BCL2 family in high-grade
MYC-associated B-cell lymphoma

Giulio Donati, Micol Ravà, Marco Filipuzzi, Paola Nicoli, Laura Cassina, Alessandro Verrecchia, Mirko Doni, Simona Rodighiero, Federica Parodi, Alessandra Boletta, Christopher P. Vellano, Joseph R. Marszalek, Giulio F. Draetta and Bruno Amati

**Fig. S1. Variable association of the MYC-V1 and OxPhos gene signatures with patient survival in DLBCL.** Kaplan-Meier survival curves for R-CHOP treated DLBCL patients from the indicated cohorts [3-5, 22-24] stratified according to the expression of genes in **(A)** Hallmark MYC-V1 or **(B)** OxPhos gene sets. The plots compare the survival of patients grouped in the top (HIGH) and bottom (LOW) tertiles, with the numbers of patients in each group given in parenthesis. For the study of Lenz et al. [22], only R-CHOP-treated patients were analyzed, excluding different treatments; in Sha et al. [23] (REMoDL-B trial), about 50% of the patients were treated with bortezomib in addition to R-CHOP, and were included in our analyses.

**Fig. S2. MycER expression and activation in the B-lymphoid cell lines FL5.12 and Ba/F3.** Following transduction with a MycER-expressing retrovirus and selection of resistant cell pools (FL^MycER^ and BaF^MycER^), the constitutively expressed MycER chimera was activated by treatment with OHT (100 nM, 48h). (**A**) Cell cycle kinetics: cells were pulse-labeled with BrdU (33 µM, 20 minutes), chased in BrdU-free medium and harvested at the indicated time points. The profiles of OHT-treated and untreated cells (red and black dots, respectively) are overlaid, revealing undistinguishable progression kinetics. (**B**) Percentage of live cells following 48h OHT treatment. Error bars: SD (n=3). (**C**) RT-PCR quantification of endogenous *MYC* and known MYC-induced mRNAs, normalized to *Tbp*. (**D**) Immunoblot analysis with a MYC-specific antibody, detecting both endogenous mouse MYC and exogenous MycER, as indicated. Vinculin was used as loading control. (**E**) Volcano plot showing fold change (FC, Log2 value) against q-value (–log10) for each mRNA in OHT-treated (100 nM, 72h) vs. untreated FL^MycER^ cells. The threshold of statistical significance for calling Differentially Expressed Genes (DEGs; q value < 0.05) is identified by the dotted line. (**F**) 10 most significantly enriched upstream regulators identified from OHT-responsive DEGs. Note that in this analysis the ISR regulator EIF2AK3 was listed further down in 29^th^ position and with a z-score of 0.056, indicating that MycER activation alone did not significantly induce this pathway in FL^MycER^ cells. (**G**) Mitochondrial stress test profiles on parental FL5.12 cells do not show the OHT-induced increases in basal respiration and mitochondrial ATP production seen in FL^MycER^ cells (Fig. 2B). Unexpectedly, here OHT-treated cells showed a moderate decrease in maximal respiratory activity relative to untreated controls (p < 0.0001): this might be due either to an unforeseen off-target effect of OHT, or to variability between parallel cultures in this experiment.

**Fig. S3. Effects of MycER activation, IACS-010759 and other pharmacogenetic interactions in B-cells.** (**A**) Percentage of live cells and Proliferation index in parental FL5.12 cells: to be compared with the data on FL^MycER^ cells in Fig. 2C-D. (**B**) Percentage of live cells (left) and Proliferation index (right; as defined in Fig. 2D) following the indicated treatments in Ndi1-expressing FL^MycER^ cells. (**C**) Percentage of live FL^MycER^ and BaF^MycER^ cells following sequential treatment with OHT (100 nM, 48h) and rotenone (48h) at the indicated concentrations. (**D**) As in (C), following sequential treatment with OHT and IACS-010759 (135 nM, 48h) at the indicated glucose concentrations. (**E**) Percentage of live cells and Proliferation index in FL^MycER^ and BaF^MycER^ cells treated with OHT and IACS-010759 (135 nM) for 48h in standard growth medium, or medium supplemented with either aspartate (Asp; 10 mM), or a combination (A/H/U) of adenine (150 µM), hypoxantine (150 µM), and uridine (400 µM). Error bars in A-D: SD (n=3). (**F**) RNA-seq profiling of the response to IACS-010759 (135 nM, 24 hours), either with (Y-axis) or without (X-axis) pre-treatment with OHT (100 nM, 48h). Black dots mark transcripts called as DEGs (q value < 0.05) in only one condition, and red dots those called in both. (**G**) Variation of known ISR-regulated mRNAs upon IACS-010759 treatment in cells grown with or without OHT from the RNA-seq data.

**Fig. S4. IACS-010759 kills FL^MycER^ cells by apoptosis.** FL^MycER^ cells were primed with OHT and treated with IACS-010759 (135 nM, 48h, unless otherwise indicated). (**A**) DAPI and TRITC-Agglutinin staining. In the IACS-010759-treated sample, note the presence of nuclei with condensed and fragmented chromatin, or faintly stained due to DNA loss. Scale bars: 10 µm. (**B**) Immunoblot analysis showing the cleaved (c-PARP) and uncleaved forms of PARP. Vinculin was used as loading control. (**C**) Caspase 3/7 activity in FL^MycER^ cells treated with OHT, IACS-010759 and/or 20 µM Z-VAD-FMK (48h) as indicated. (**D**) Representative images of DAPI and cytochrome c staining (left) and relative quantification of the cytochrome c cellular area (right). *P < 0.0001 (Student’s t test). Scale bars: 10 µm. **(E**) Z-VAD-FMK does not prevent killing of FL^MycER^ cells by IACS-010759 and venetoclax in the absence of OHT. **(F, G**) Mitochondrial stress test profiles of control and BCL2-overexpressing FL^MycER^ cells, primed with OHT and treated with IACS-010759 (135 nM) and/or venetoclax (100 nM) for 24 hours, as indicated. (**H**) Percentage of live Bax/Bak knockout FL^MycER^ cells following treatment with IACS-010759 (135 nM) and/or venetoclax (100 nM), as indicated. Error bars: SD (n=3).

**Fig. S5. Effects of IACS-010759 and BH3-mimetics on human lymphoma cells.** (**A**) Percentage of live cells after treatment (24h) of the human DHL cell lines Karpas 422 and SU-DHL-4 with IACS-010759 (135 nM) and/or venetoclax (VTX, 100 nM), as indicated. *P < 0.0001. (**B**) Immunoblot analysis of total, phosphorylated (P-) eIF2a and ATF4 in the indicated DHL cell lines, treated with IACS-010759 (135 nM) for 24 hours. Vinculin was used as loading control. DOHH-2 lysates are from a different experiment, generated during the revision of our manuscript. (**C**) Summary of the response of subcutaneous SU-DHL-6 tumors to treatment with IACS-010759 and/or venetoclax, as indicated, in two independent experiments. Detailed progression curves for experiment 1 are shown in Figure 5C. Partial and Complete Regression at day 19 (one week after the end of treatment) were defined as ≤50% and ≤10% of the starting tumor volume (day 0), respectively.

**Fig. S6. Whole body imaging of PDX-engrafted mice treated with IACS-010759 and/or venetoclax.** Whole body images (ventral view) of PDX-bearing mice at day 14 (referred to Fig. 5E).

**Table S1. MYC- and OxPhos-associated gene signatures are correlated in DLBCL.** Gene expression datasets from the DLBCL patient cohorts profiled in Chapuy et al. 2018 [4], Ennishi et al. 2019 [24], Lenz et al. 2008 [22], Reddy et al. 2017 [3], Schmitz et al. 2018 [5] and Sha et al. 2019 [23] were analyzed to define the pairwise correlations between each of the indicated MYC- and OxPhos-related gene expression signatures (Genes sets 1, 2), taken from the Hallmark collection (MYC-V1, MYC-V2 and Hallmark-OxPhos) and the CCC model (CCC-OxPhos). Within each cohort (indicated on the left), the rows represent each of the six pairwise comparisons (Gene set 1 and 2), ordered by decreasing Pearson correlation, with the indication of their statistical significance (p-value). Genes between shared between the compared signatures (Table S2) were excluded from the calculation of each correlation.

| **Gene set 1 (nr. of genes)** | **Gene set 2 (nr. of genes)** | **Nr. of overlapping genes** |
| --- | --- | --- |
| Hallmark-OxPhos (200) | MYC-V1 (200) | 11 |
| Hallmark-OxPhos (200) | MYC-V2 (58) | 1 |
| Hallmark-OxPhos (200) | CCC-OxPhos (56) | 18 |
| CCC-OxPhos (56) | MYC-V1 (200) | 3 |
| CCC-OxPhos (56) | MYC-V2 (58) | 0 |
| MYC-V1 (200) | MYC-V2 (58) | 18 |

**Table S2. Genes shared among the MYC- and OxPhos-related signatures**

| **Target protein** | **Source** | **Clone** | **Vendor** | **Cat #** |
| --- | --- | --- | --- | --- |
| Vinculin | mouse | hVIN-1 | Merck Life Science | V9131 |
| Myc | mouse | Y69 | Abcam | ab32072 |
| PARP | rabbit | polyclonal | Cell Signaling Technology | 9542 |
| BCL2 | rabbit | D17C4 | Cell Signaling Technology | 3498 |
| Bcl-XL | rabbit | E18 | Abcam | ab32370 |
| Bax | mouse | E63 | Merck Life Science | ab32503 |
| Bim | mouse | C34C5 | Cell Signaling Technology | 2933 |
| PUMA a/b | rabbit | polyclonal | Santa Cruz Biotechnologies | sc-28226 |
| Phospho-eIF2a (S51) | rabbit | D9G8 | Cell Signaling Technology | 3398 |
| eIF2a | rabbit | D7D3 | Cell Signaling Technology | 5324 |
| ATF4 | rabbit | D4B8 | Cell Signaling Technology | 11815 |
| CHOP | mouse | L63F7 | Cell Signaling Technology | 2895 |
| Pck2 | rabbit | polyclonal | Cell Signaling Technology | 6924 |
| Gpx1 | rabbit | polyclonal | Abcam | ab22604 |
| Mcl-1 | rabbit | polyclonal | Santa Cruz Biotechnologies | sc-819 |

**Table S3. Antibodies used for immunoblot analysis**

| **Target gene** | **NCBI gene ID** | **Forward primer sequence** | **Reverse primer sequence** |
| --- | --- | --- | --- |
| Tbp | 21374 | TCAAACCCAGAATTGTTCTCC | TTCAAATGCTTCATAAATCTCTGC |
| Myc | 17869 | TTTTTGTCTATTTGGGGACAGTG | CATCGTCGTGGCTGTCTG |
| St6Galnac4 | 20448 | TGGTCTACGGGATGGTCA | CTGCTCATGCAAACGGTACAT |
| Rrp9 | 27966 | TTCTAGCGGACGCGATAAAC | ACTTCTGCAACCTGCCTCTC |

**Table S4. Primers for mRNA quantification by qPCR**

| **Target gene** | **NCBI gene ID** | **sgRNA target sequence** | **Locus** |
| --- | --- | --- | --- |
| Ddit3 | 13198 | TCAGCTGCCATGACTGCACG | chr10:127295332-127295351 |
| Ddit3 | 13198 | CTGTCCTCAGATGAAATTGG | chr10:127295425-127295444 |
| Bax | 12028 | AGCGAGTGTCTCCGGCGAAT | chr7:45466013-45466032 |
| Bax | 12028 | AGTTTCATCCAGGATCGAGC | chr7:45466106-45466125 |
| Bak | 12018 | TCATCGCAGCCCACCTTCGG | chr17:27025810-27025829 |
| Bak | 12018 | CTGGTGTGCGCACATGCGCA | chr17:27025715-27025734 |

**Table S5. Genomic targets of the sgRNAs used for CRISPR-Cas9 gene knockout**

| **Target gene** | **NCBI gene ID** | **Forward primer sequence** | **Reverse primer sequence** | **Amplicon** |
| --- | --- | --- | --- | --- |
| Ddit3 | 13198 | CCCATGCCCTTACCTATCGT | AGGAGAGGCATACAAACCCC | chr10:127295211-127295680 |
| Bax | 12028 | AACATTCTGCTCCTCTCCCC | CAGAGCACCGCCTACTAGAA | chr7:45465728-45466162 |
| Bak | 12018 | AGGTCACACATCACTACCCG | AATGCCATTCCCTGTCCACA | chr17:27025495-27025949 |

**Table S6. Primers for PCR analysis of CRISPR-Cas9 knockout clones**
